# A silicon membrane microfluidic oxygenator for use as an artificial placenta with minimal anticoagulation

**DOI:** 10.1002/btm2.70037

**Published:** 2025-07-12

**Authors:** David G. Blauvelt, Nicholas C. Higgins, Anne Hesek, Bianca N. De, Nathan Wright, Prasad Nithianandam, Charles Blaha, Jarrett Moyer, Benjamin W. Chui, Francisco J. Baltazar, Shuvo Roy

**Affiliations:** ^1^ Department of Pediatrics Nemours Children's Health Wilmington Delaware USA; ^2^ Department of Pediatrics Sidney Kimmel Medical College at Thomas Jefferson University Philadelphia Pennsylvania USA; ^3^ Department of Pediatrics University of California San Francisco California USA; ^4^ Department of Bioengineering and Therapeutic Sciences University of California San Francisco California USA; ^5^ Department of Research Nemours Children's Health Wilmington Delaware USA; ^6^ School of Medicine University of California San Francisco California USA

**Keywords:** artificial placenta, extracorporeal life support, extracorporeal membrane oxygenation, microfabrication, microfluidics, prematurity, silicon membranes

## Abstract

Extreme prematurity carries a high burden of morbidity and mortality. The artificial placenta is an emerging therapy that has the potential to improve outcomes in these patients. However, current devices in development are limited by inadequate hemocompatibility, a major barrier to the translation of the artificial placenta into humans. Here, we present a novel microfluidic oxygenator that is comprised of a stacked array of semiconductor silicon membranes and operates with minimal anticoagulation (activated clotting time = 120–180 s). We describe the design, construction, and testing of two generations of prototypes. Our Generation 2 Device had an oxygen transfer of 1.51 ± 0.25 volume % (mean ± standard error). Computational fluid dynamics (CFD) modeling demonstrated favorable blood flow properties, including laminar flow, no stasis or recirculation, and optimal wall shear stress. In vivo testing in a 6 hour neonatal swine model showed that the silicon membrane oxygenator could operate with low‐dose anticoagulation with minimal clot formation. Furthermore, the oxygenator had no significant effect on markers of animal health, including inflammation (white blood cell count), coagulation (platelet count, prothrombin time), or hemolysis (hematocrit, plasma free hemoglobin). This study represents a key advance toward developing an anticoagulation‐free oxygenator and ultimately bringing artificial placenta technology to patients.

**Translational Impact Statement**

An artificial placenta is an oxygenator device that may improve outcomes in extremely preterm infants, but current technology is limited by inadequate hemocompatibility. In this study, we developed a microfluidic oxygenator designed to operate with minimal anticoagulation. It features a stacked array of flat plate semiconductor silicon membranes, which enable a blood flow path with flow dynamics designed to reduce clot formation. Our benchtop and in vivo testing demonstrate promising oxygen transfer and hemocompatibility. Future work will aim to translate this technology to clinical use. A silicon microfluidic oxygenator enables efficient, low‐anticoagulation oxygenation, advancing artificial placenta technology for preterm infants.

## 1 INTRODUCTION

Premature birth carries a high burden of mortality. Although neonatal critical care has improved the outcomes of premature infants over the last few decades, when born before 22 weeks of gestation, infants are considered non‐viable and cannot survive outside the womb.^1^ In the United States, for those born between 22 and 24 weeks, only one third survive hospital discharge.^2^ A major driver of the poor outcomes in these patients is the reliance on invasive mechanical ventilation to support gas exchange. Although mechanical ventilation is necessary to prevent immediate respiratory failure and death, it can arrest normal lung development and inflict damage on the premature lung units.^3^, ^4^, ^5^

A potential alternative to mechanical ventilation is the “artificial placenta,” an adaptation of extracorporeal life support (ECLS) technology to extremely preterm infants. In the artificial placenta, gas exchange is performed outside the body via an external oxygenator device, thereby sparing the lungs from the harms of mechanical ventilation. Several artificial placenta devices have shown promise in animal models, enabling long‐term survival.^6^, ^7^, ^8^, ^9^

Unfortunately, a key drawback of current artificial placenta devices in development is their reliance on standard ECLS hollow fiber membrane oxygenators, which have several limitations. Hollow fiber membrane oxygenators typically have large priming volumes that may exceed the entire blood capacity of the infant.^10^ Additionally, they require high doses (i.e., “therapeutic,” as opposed to “prophylactic” drug levels) of systemic anticoagulation to prevent thrombosis. While anticoagulation can reduce device clotting, it also leads to severe patient bleeding. In hollow fiber membrane devices used for neonatal ECLS, 12% of patients develop intracranial hemorrhage, which is frequently fatal.^11^ This risk is increased in premature infants due to their fragile cerebral vasculature. Even without anticoagulation, more than 50% of infants born at 24 weeks develop intracranial hemorrhage from spontaneous cerebral vessel rupture.^12^ Consequently, anticoagulation for an artificial placenta is a significant risk for extremely preterm patients. In fact, a gestational age of less than 34 weeks is a contraindication to ECLS due to this risk.^13^ Therefore, extremely preterm infants have no options for extracorporeal gas exchange and must rely on injurious mechanical ventilation. The need for high‐dose systemic anticoagulation in hollow fiber membrane oxygenators is a major barrier preventing clinical translation of artificial placenta devices.^14^, ^15^

An important contributor to the need for anticoagulation in hollow fiber membrane oxygenators is their limited control over blood flow dynamics. As blood flows through a hollow fiber membrane oxygenator, it must navigate around a woven mesh of hollow fibers.^16^ Although this can improve gas transfer efficiency by promoting mixing, the heterogeneous flow dynamics can lead to high shear forces, recirculation, and stasis, all of which promote thrombosis.^17^, ^18^, ^19^

Here, we present a novel microfluidic artificial placenta oxygenator designed to operate with minimal anticoagulation. A key difference between our oxygenator and existing hollow fiber membrane oxygenators is the design of the blood flow path, which is optimized for hemocompatibility. Our strategy is enabled by the development of microfluidic semipermeable silicon membranes, which are composite membranes with a semiconductor silicon backbone and a thin, gas‐permeable elastomer layer.^20^, ^21^, ^22^ We previously showed in proof‐of‐concept miniature oxygenators that these membranes can perform gas exchange.^22^ In this study, we expand upon our prior work by increasing the scale of the devices and evaluating their hemocompatibility. In our oxygenators, instead of blood flowing heterogeneously around a bundle of fibers, blood flows smoothly between flat plates. We take advantage of the unique rigidity of the flat plate silicon membranes to create an oxygenator with a precisely stacked array of membranes. After constructing and testing an early prototype (Generation 1 Device), we use those results to inform the design of an advanced prototype (Generation 2 Device). Blood testing of the Generation 2 Device demonstrated oxygen flux close to that required for a periviable preterm infant. Using computational fluid dynamics (CFD) modeling, we demonstrated favorable properties of laminar blood flow as well as avoidance of both stasis and high shear forces. Finally, we evaluated its hemocompatibility in vivo using an extracorporeal pig model, demonstrating that the silicon membrane oxygenator can operate for at least 6 h with minimal anticoagulation.

## 2 RESULTS

### 2.1 Design considerations for a silicon membrane artificial placenta oxygenator

To support periviable infants, the target design parameters (Table S1, Supplementary Material) need to be tailored to 20–23 week gestational age infants (approximately 330–570 g).^23^ Unlike ECLS, which incorporates an external pump, several groups have had success with pumpless circuits connected to the umbilical circulation.^9^, ^24^ Key considerations for a pumpless artificial placenta include achieving stable, long‐term cannulation of the umbilical vessels—a technically challenging task due to vasospasm^25^—and using a low resistance oxygenator with a pressure drop that closely matches the pressure differential between the umbilical artery and vein (approximately 30 mmHg).^9^, ^26^ Additionally, the target flow rate should match in utero placental flow rate (approximately 200 mL/kg/min = 67–133 mL/min) to ensure an appropriate balance of flow to the artificial placenta and the body, which are parallel flow systems.^9^, ^26^, ^27^ Oxygen flux will need to match the expected oxygen consumption (6 mL/kg/min), which equates to an oxygen transfer rate of 3 volume percent (vol%), or 2.0–3.5 mL/min in absolute flux.^27^ The required carbon dioxide removal is approximately 0.8 times that of oxygen consumption (2.4 vol% or 1.6–2.8 mL/min). Another important consideration is wall shear stress (WSS). Low WSS (<1 Pa) has been associated with thrombosis formation due to stasis,^28^ while supraphysiologic WSS (>3.6 Pa) can induce platelet activation, and very high WSS (>500 Pa) can cause hemolysis.^17^, ^29^, ^30^, ^31^ Most importantly, the oxygenator needs to operate with minimal anticoagulation. Ideally, it would operate clot‐free without anticoagulation. However, some studies have shown that prophylactic dose systemic heparin may be safe, even in extremely preterm infants, though these studies are several decades old and did not incorporate comprehensive coagulation profiling.^32^, ^33^, ^34^

### 2.2 First generation device

**FIGURE 1:**
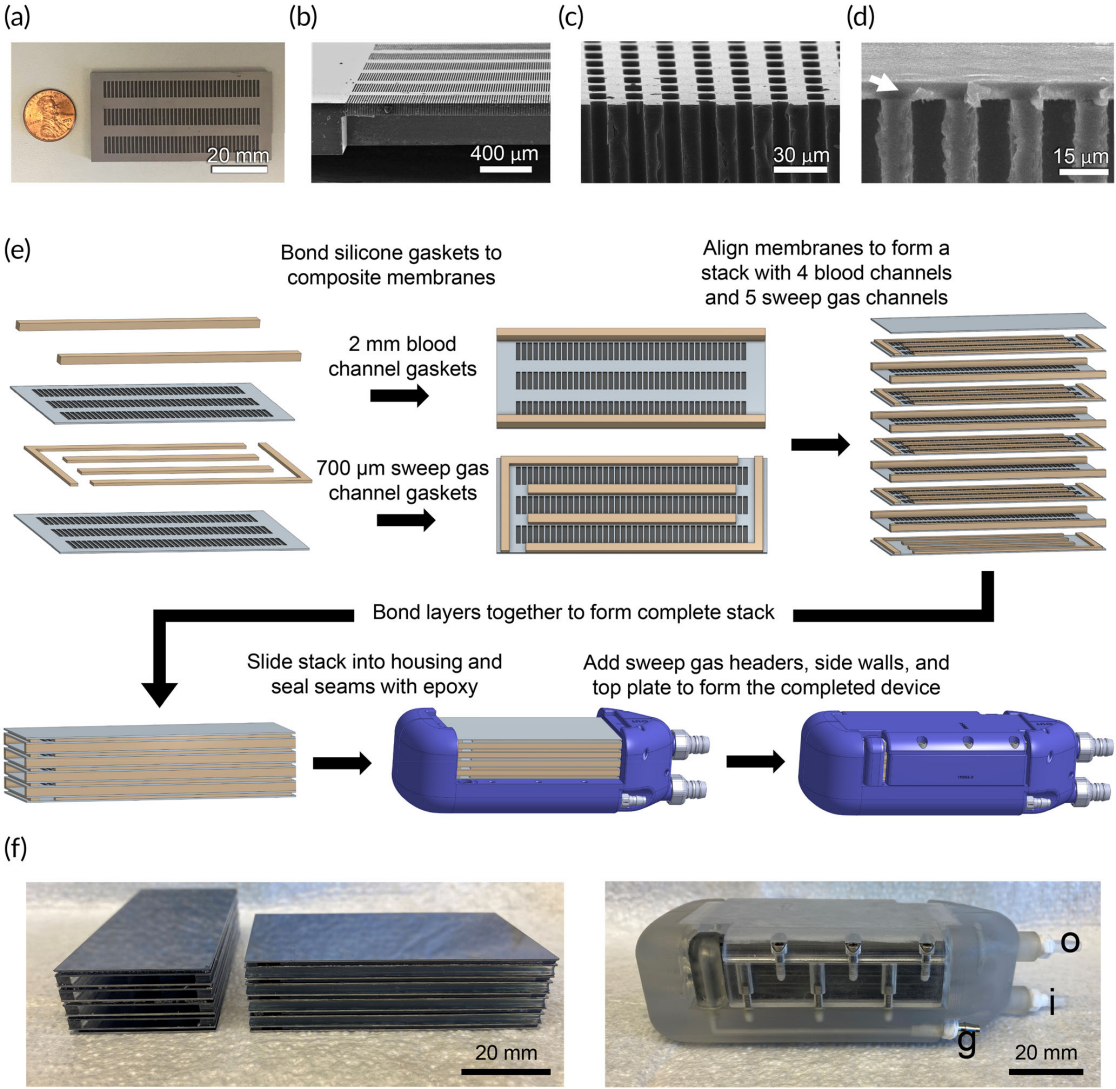
Generation 1 Device assembly. (a) En face photographic view of the silicon membrane. (b–d) Scanning electron micrographs showing (b) an oblique view of the pores and large windows, (c) a cross‐sectional view of a cut through uncoated pores, and (d) a cross‐sectional view of a cut through pores bonded to a 5 μm layer of PDMS (arrow). (e) The membrane stack is formed by bonding silicone gasket spacers, which form the blood and sweep gas channels. The completed stack is composed of 8 total membranes bounded by 2 solid silicon end pieces, which forms 4 blood channels and 5 sweep gas channels. The stack is placed into the housing along guide rails and sealed with medical‐grade epoxy. Sweep gas headers are added to direct the sweep gas to the correct channels. Side and top plates provide mechanical support. (f) Photographs of the completed stacks (left), showing the alternating blood and sweep gas channels, and assembled device (right) after sealing the stack into the 3D printed housing. i = blood inlet, o = blood outlet, g = sweep gas connector.

Our Generation 1 Device was composed of microfluidic silicon membranes and a 3D‐printed housing. The silicon membranes were fabricated by etching microscopic pores into semiconductor silicon, followed by bonding a thin (5 μm) layer of gas‐permeable polydimethylsiloxane (PDMS) to the pore‐containing silicon surface (Figure S1, Supplementary Material). The resulting composite membranes were evaluated with scanning electron microscopy, which demonstrated excellent uniformity of both the pores and PDMS thickness (Figure 1a–d).

Next, silicon membranes were arranged into a stack using PDMS gaskets, which sealed the long edges of the channels, defined the channel heights, and helped provide mechanical support (Figure 1e). The stack was placed into a 3D‐printed housing using rails in the housing to guide the alignment of the channels. The seams of the stack were sealed with epoxy to prevent leaking. Finally, side walls and a top plate were screwed into place to add mechanical support, forming the completed oxygenator (Figure 1f). Device parameters are summarized in Table S2, Supplementary Material.

**FIGURE 2:**
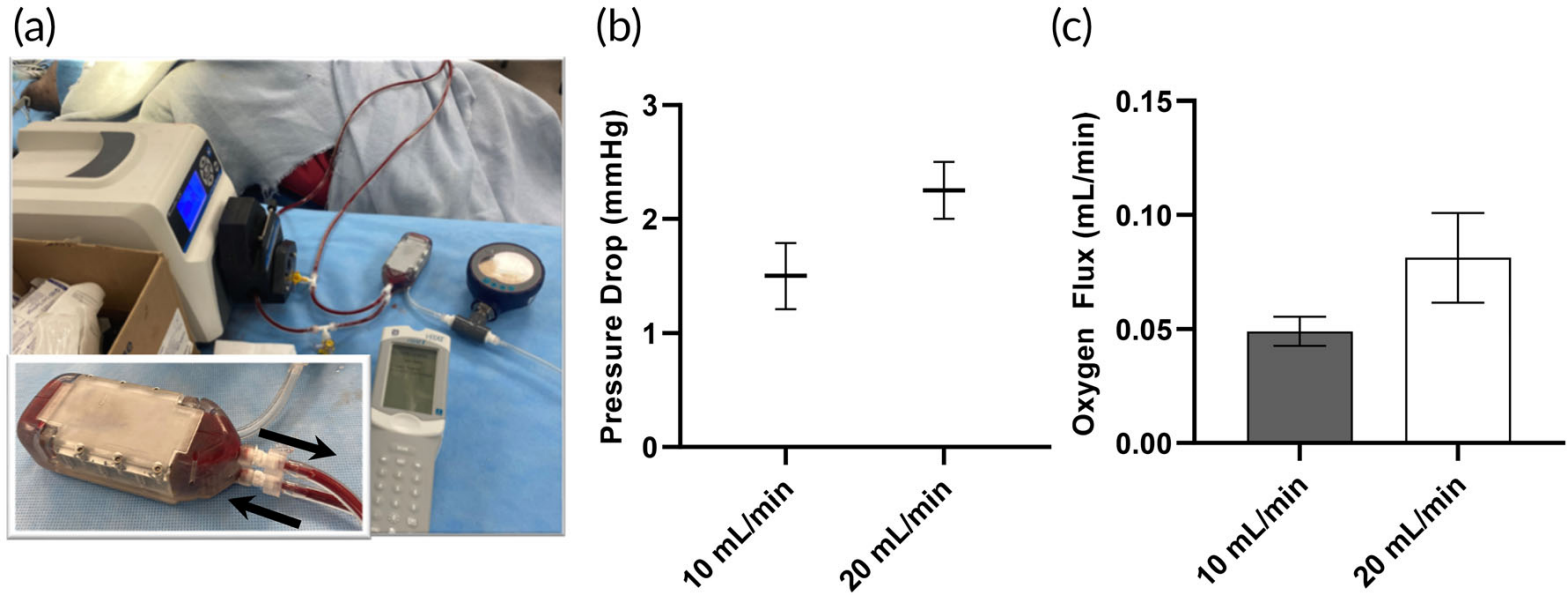
Generation 1 Device testing results. (a) Photographs of the device undergoing testing (b) Pressure drop measurements at 10 and 20 mL/min of blood flow. (c) Oxygen flux at 10 and 20 mL/min of blood flow. For all experiments, *n* = 4 devices, 6 measurements per device per condition. Data is presented as mean ± standard error.

Four oxygenator prototypes were assembled for testing using an extracorporeal porcine model (Figure 2). Venous blood was pumped through the device at 10 and 20 mL/min using a peristaltic pump. Baseline venous blood parameters (mean ± standard error of the mean [SEM]) were: pCO_2_ = 43.3 ± 0.7 mmHg, pO_2_ = 44.5 ± 0.7 mmHg, sO_2_ = 80.8 ± 0.6%, Hemoglobin = 7.1 ± 0.1 g/dL. Pressure drop was 1.50 ± 0.29 mmHg (mean ± SEM, *n* = 4 devices) and 2.25 ± 0.25 mmHg for 10 and 20 mL/min of blood flow, respectively. Oxygen saturation (%) increased by 4.8 ± 0.4 (mean ± SEM, *p* < 0.001 [paired t‐test], n = 4 devices, 6 measurements per device per flow rate) and 4.0 ± 1.3 (p < 0.001) for 10 and 20 mL/min, respectively (Table S3, Supplementary Material). Oxygen flux (mL/min) was 0.049 ± 0.006 at 10 mL/min blood flow and 0.081 ± 0.020 at 20 mL/min (mean ± SEM, n = 4 devices, 6 measurements per device per flow rate). Carbon dioxide flux was not evaluated in our initial prototype.

### 2.3 Design improvements and second generation device

**FIGURE 3:**
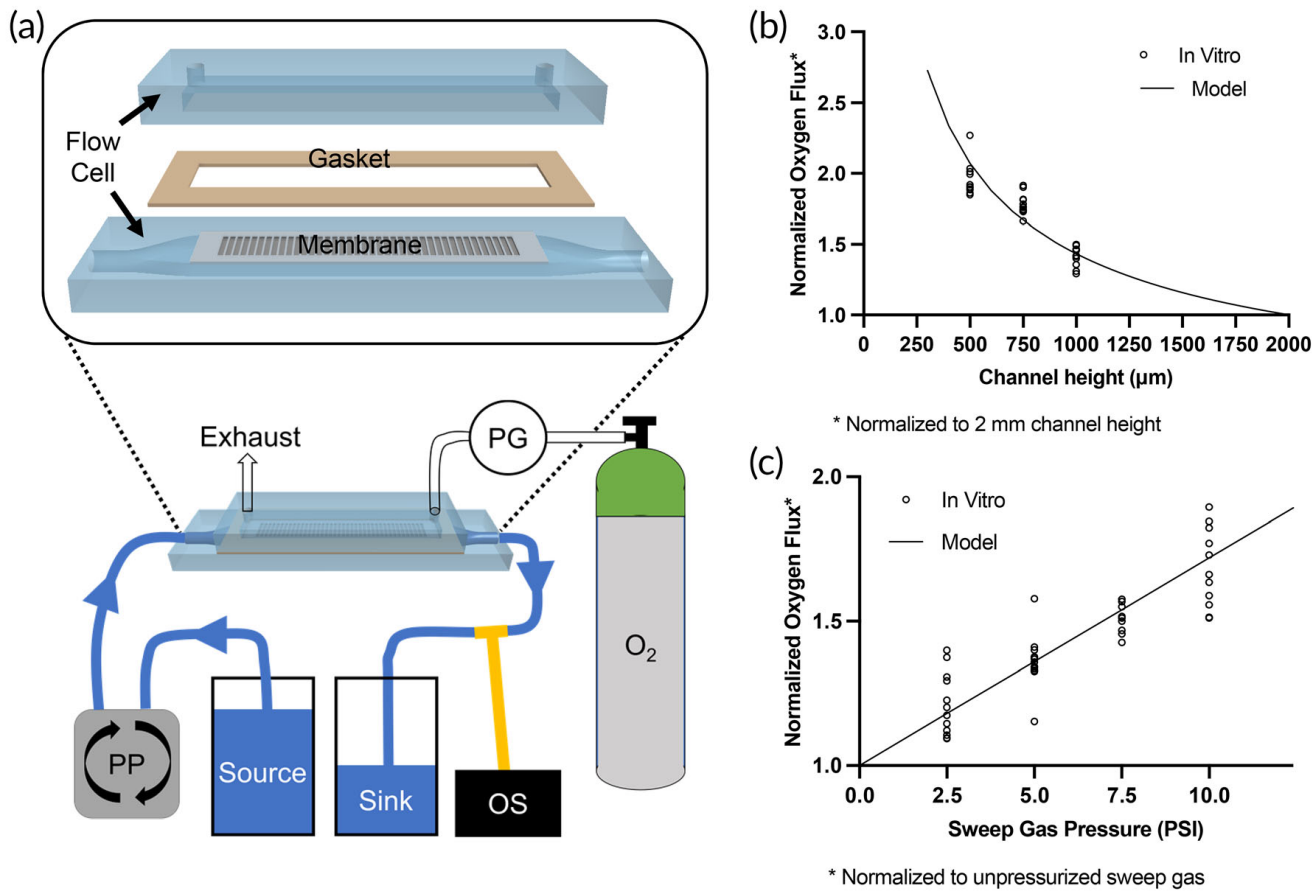
Gas transfer as a function of channel height sweep gas pressure. (a) A benchtop mock loop circuit was used to investigate the effect of channel height and sweep gas pressure on oxygen flux. The membrane was housed in a custom‐machined flow cell and sealed with a silicone gasket. Water was pumped through the circuit by a peristaltic pump (PP), and sweep gas pressure was measured with a pressure gauge (PG). Changes in oxygen concentration were measured with an optical sensor (OS). (b) Relative oxygen flux as the channel height is reduced from 2 mm. Benchtop measurements demonstrate excellent alignment with modeled predictions. (c) Relative oxygen flux as sweep gas is pressurized.

Although our initial prototype met our pressure drop requirements, it fell short of our target gas exchange parameters. We therefore sought to identify design changes that could improve gas exchange efficiency. Using the data from our Generation 1 Device testing, we adapted a computational mass transfer model (custom coded in MATLAB) to predict oxygen flux based on changes in design parameters.^35^ We further validated our mass transfer predictions using a benchtop flow cell (Figure 3a). A linear regression of the modeling data versus benchtop measurements showed excellent agreement (slope = 0.97, *r* ^2^ = 0.99) (Figure S2, Supplementary Material). Although measuring oxygen transfer into water is not a perfect substitute for blood measurements, we have found that design changes that increase oxygen flux into water have a similar relative effect on flux into blood.^22^ Additionally, water testing offered two advantages over blood. First, water does not foul the membranes, which enabled efficient, non‐destructive testing of iterative design changes. Second, oxygen sensors that detect dissolved oxygen in water are more sensitive to small changes in oxygen flux compared to blood gas analyzers, which have higher measurement variability.

Using this strategy, we found that decreasing the channel height (i.e., the distance between parallel membranes) and increasing the sweep gas pressure had dramatic effects on oxygen flux (Figure 3b,c). Our Generation 1 Device featured a channel height of 2 mm and used unpressurized sweep gas. By decreasing the channel height from 2 to 0.5 mm we could expect to improve efficiency by 2.1‐fold. In addition, by pressurizing the sweep gas we found an improvement in flux that increased linearly by 7.2% per PSI. Furthermore, these design changes affected flux in an independent manner, such that implementing both changes would have a multiplicative effect.

**FIGURE 4:**
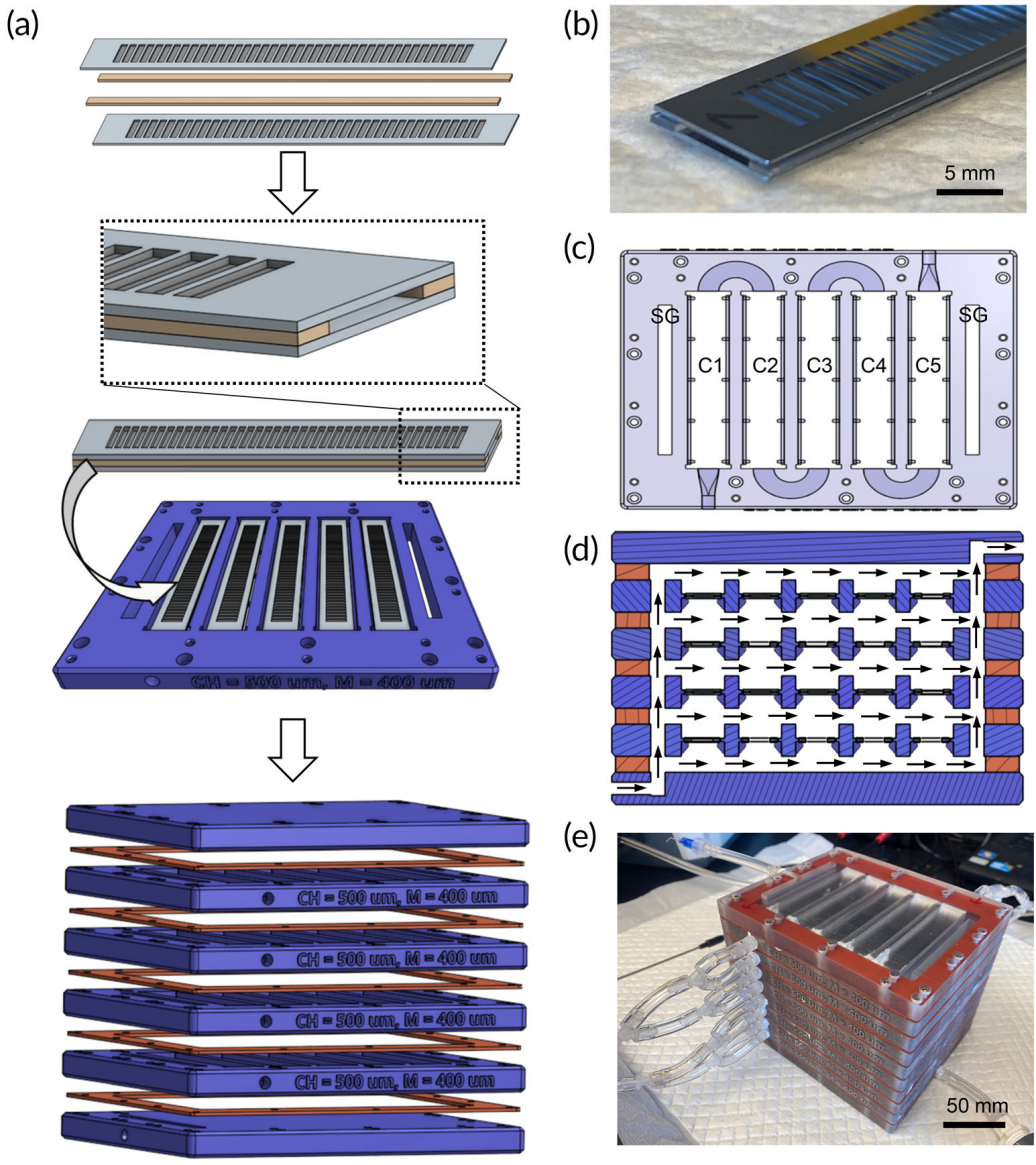
Generation 2 Device assembly. (a) Membrane channels are formed by stacking two membranes between a 500 μm thick silicone gasket. The blood channel is thus bounded on the top and bottom by a membrane and by the gaskets along the side. The stack is sealed into a 3D printed housing that directs blood into the channel. The completed device is created by stacking multiple layers, each separated by 1 mm thick silicone gaskets, which enables sweep gas to freely travel between the layers. (b) Photograph of a single silicon membrane stack. (c) Sectional view through the 3D printed layer showing the membrane channels (C1‐5) and the two sweep gas (SG) distribution channels. (d) Sectional view through the device showing the path of the sweep gas through multiple layers (arrows). (e) Photograph of a completed device composed of eight layers.

Based on this work, we developed our Generation 2 Device (Figure 4). The device was a multilayered device that could be scaled to match the size of the patient. The assembly process is illustrated in Figure 4a. Each individual layer contained 5 membrane stacks. A stack was composed of two silicon membranes separated by two PDMS gaskets that were bonded to the membrane long edges (Figure 4b). The gaskets were 500 μm thick, thus creating a 500 μm channel height. Each stack was placed into a 3D printed housing that interfaced with the membranes to form a continuous blood channel (Figure 4c). The multilayered device was then assembled with 1 mm thick silicone gaskets separating layers. The gaskets enabled sweep gas to move freely between layers but sealed the device from the outside (Figure 4d). The final device was then created by attaching a tubing manifold to distribute blood to each channel in a parallel flow manner (Figure 4e). The device parameters are summarized in Table S2, Supplementary Material.

**FIGURE 5:**
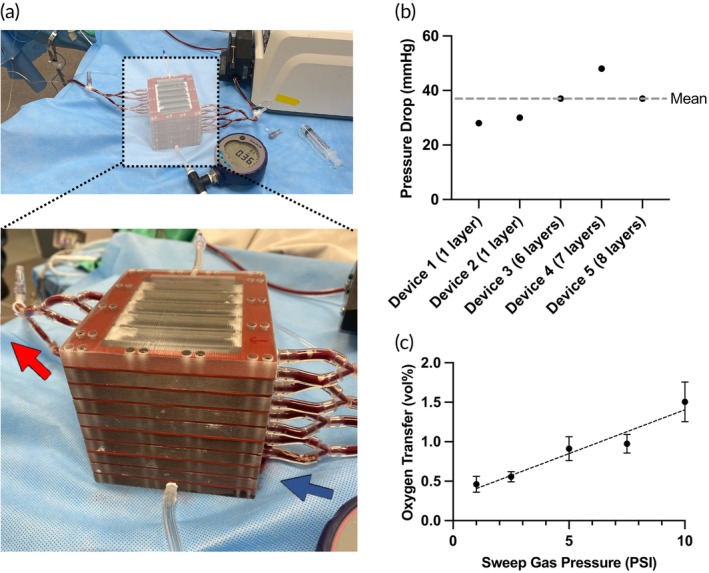
Generation 2 Device testing. (a) Photograph of a 8‐layered device undergoing testing. Arrows denote blood flow. (b) Pressure drop for the five devices tested. The flow rate of the devices varied based on the number of layers such that the total flow rate was 10 mL/min per layer. Devices 1 & 2: 1 layers, 10 mL/min; Device 3: 6 layers, 60 mL/min; Device 4: 7 layers, 70 mL/min; Device 5: 8 layers, 80 mL/min. (c) Oxygen flux was calculated as a function of sweep gas pressure. To compare devices of different sizes run at different blood flow rates, oxygen transfer was calculated as vol%. Data is presented as mean ± standard error.

Five oxygenators were assembled for testing in an extracorporeal pig model (Figure 5a). Devices 1 and 2 were single‐layer devices, and devices 3–5 were multilayered devices with between 6 and 8 layers. The number of layers was varied to evaluate the ability of the device to scale linearly with the number of layers. All devices were run at an overall flow rate such that the flow through each layer was 10 mL/min (e.g., 80 mL/min for the 8‐layer device). Each layer had a priming volume of 1.5 mL (12 mL prime for the largest 8‐layer device +3 mL prime for the manifold). The pressure drop across the five devices varied between 28 and 48 mmHg, with an average of 36.0 ± 3.5 mmHg (mean ± SEM) (Figure 5b). Oxygen flux was measured at variable sweep gas pressures. Baseline venous blood parameters (mean ± SEM) were: pCO_2_ = 48.8 ± 1.4 mmHg, pO_2_ = 42.1 ± 0.7 mmHg, sO_2_ = 76.9 ± 0.9%, Hemoglobin = 10.6 ± 0.4 g/dL. To compare devices with different numbers of layers (and therefore different blood flow rates), we calculated the vol% of oxygen transfer, defined as the amount of oxygen flux (in mL/min) per 100 mL/min of blood flow. Oxygen transfer increased from 0.46 ± 0.10 vol% (mean ± SEM) at 1 PSI of sweep gas pressure to 1.51 ± 0.25 vol% at 10 PSI (Figure 5c). Change in inlet/outlet oxygen saturation was similar across devices with different numbers of layers and thus different flow rates (Table S4, Supplementary Material). Overall carbon dioxide transfer was 0.34 ± 0.14 vol% (mean ± SEM).

### 2.4 Computational fluid dynamics blood flow analysis

**FIGURE 6:**
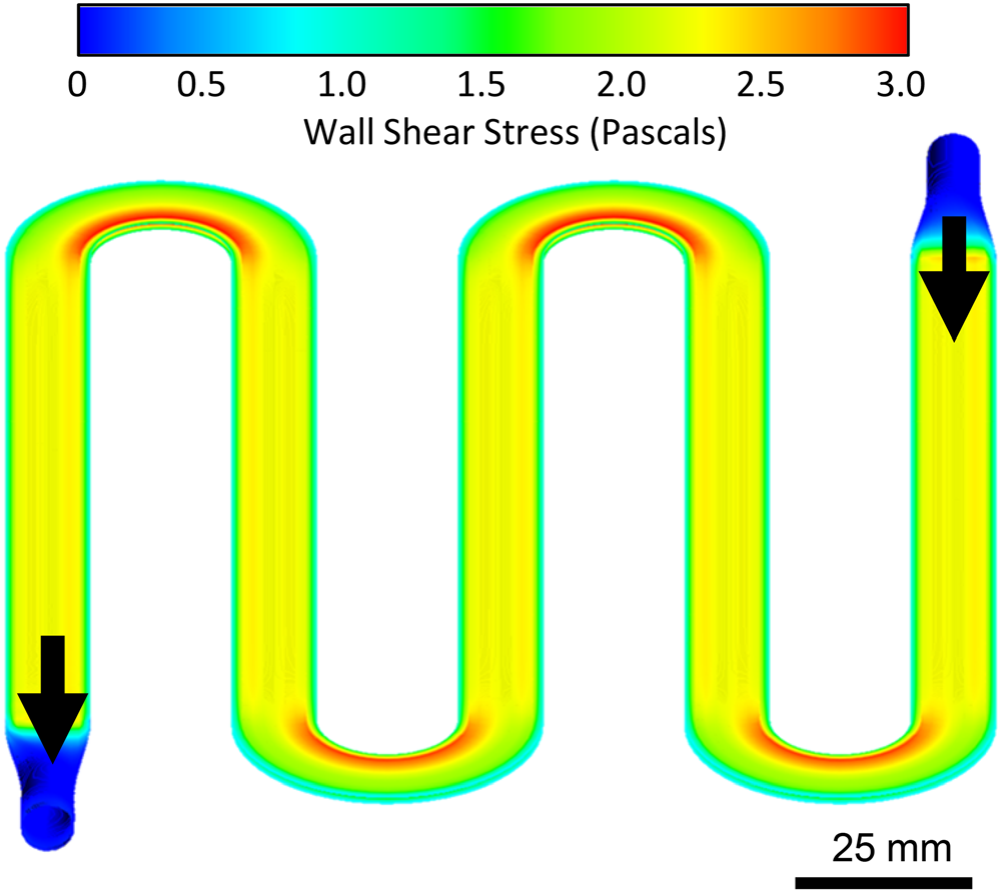
CFD modeling of wall shear stress (WSS) experienced by blood traversing a single layer of the Generation 2 Device. WSS was largely within the desired range of 1 to 3.6 Pascals, with the exception of low WSS at the inlet and outlet.

A key advantage of the silicon membrane oxygenator is the rigid flat plate membrane design, which can improve hemocompatibility by enabling a blood flow path with smooth, laminar flow. CFD modeling is an established computational tool used to evaluate the blood flow dynamics in extracorporeal oxygenators.^17^, ^36^ It has been used to identify flow features that improve hemocompatibility, including optimal WSS, no stasis, no recirculation, and no turbulence. We therefore sought to evaluate our blood flow path using CFD. The blood flow path was generated using computer‐aided design and converted into a mesh containing 40,000 hexahedral elements; the channels contained 5 elements across the 500 μm width. CFD modeling was then performed using a blood flow rate of 10 mL/min – the flow rate through each layer. The results demonstrated laminar blood flow throughout the device. The Reynolds number within the membrane bounded channels was 8.4; turbulence typically occurs at a Reynolds number greater than 2900. A streamline analysis demonstrated no areas of stasis or recirculation (Figure S3, Supplementary Material). Wall shear stress was largely within the target of >1 Pa, including throughout the main gas exchange channels (Figure 6). A notable exception to this was the inlet/outlet, which was designed to interface with standard 1/8″ barb connectors; this resulted in WSS as low as 0.1 Pa. Peak WSS was 3.0 Pa, within our target of <3.6 Pa.

### 2.5 Hemocompatibility testing

We then evaluated single‐layer oxygenators in vivo using a neonatal swine model. For this study, we used membranes consisting of transparent glass (silicon dioxide) coated with PDMS, which allowed easy visualization of clot formation. Pigs were anesthetized, and vascular cannulas were placed in the right carotid artery and jugular vein. The animals were randomized to one of three groups: no heparin, prophylactic dose heparin, and control. In the no heparin group (*n* = 4), the pigs were not anticoagulated. In the prophylactic dose group (*n* = 5), pigs were given an infusion of heparin, with a dosage titrated to maintain an activated clotting time (ACT) between 120 and 180 s. In the control group (*n* = 4), the circuit consisted of only tubing with no oxygenator, as an attempt to control for the effects of the surgery, pump, cannulas, and tubing; pigs were not anticoagulated. Blood was pumped through the circuit at a flow rate of 10 mL/min for a total of 6 h. The circuits were photographed every 2 h to quantify thrombosis. Additionally, blood was drawn every 2 h to evaluate markers of inflammation (white blood cell count), coagulation (platelet count, prothrombin time), and hemolysis (hematocrit, plasma free hemoglobin).

**FIGURE 7:**
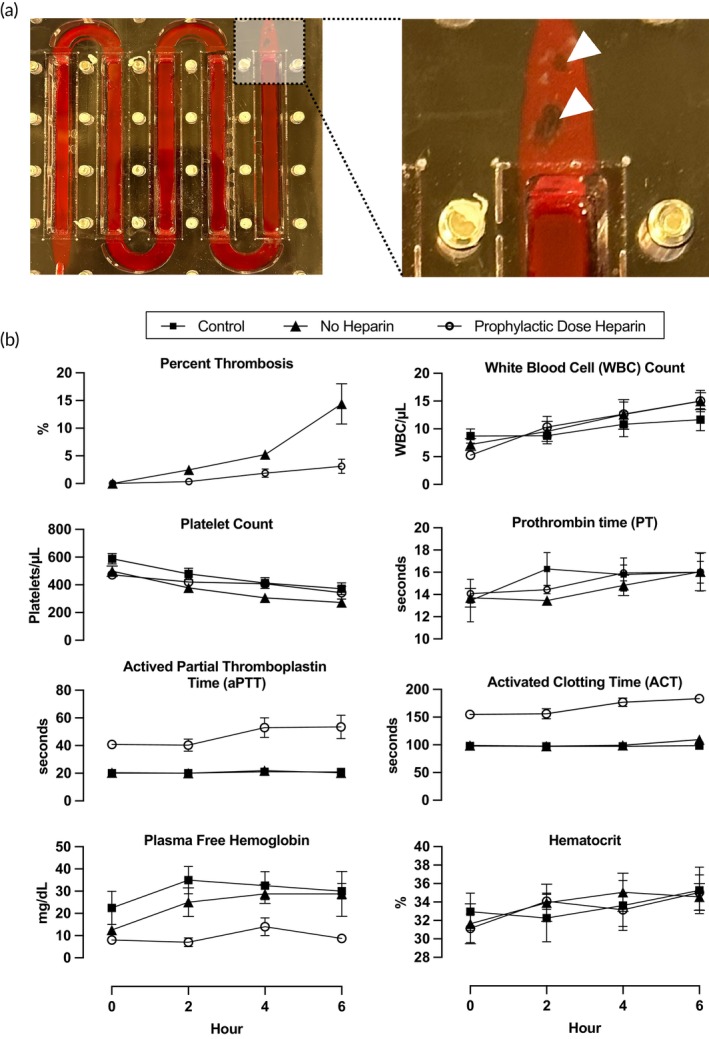
In vivo hemocompatibility studies in a neonatal pig model. (a) Photograph showing the most frequent location for thrombosis formation (arrowheads) was at the inlet/outlet. (b) Relative thrombosis and markers of animal health as a function of time. Relative thrombosis was only calculated for the device groups; the control group contained the cannulas, tubing, and connectors only. Data is presented as mean ± standard error.

In the no heparin group, 1 of the 4 devices failed after 5 h of operation due to thrombosis. None of the five devices in the prophylactic dose heparin group failed. The most common site for clot formation was at the inlet (Figure 7a); all the devices in the no heparin group and 4 of 5 devices in the prophylactic dose heparin group developed clots at the inlet. This was also noted to be the first site of thrombus formation, which then led to clot propagation to other areas. Another frequent location of thrombosis was along the channel walls, especially on the outer edge of the flow curves. This was seen in all the devices in the no heparin group and 3 of the devices in the prophylactic dose heparin group. The clot burden of each device was quantified using photographs taken at 2 h intervals. At the end of 6 h, the total clot burden as a percentage of the total flow path was 14.4% ± 3.6% (mean ± SEM) in the no heparin group and 3.1% ± 1.3% in the prophylactic dose heparin group (Figure 7b).

Markers of animal health were also measured at 2 h intervals throughout the study (Figure 7b). ACT levels were measured to confirm that they were in range based on the assigned group. The average ACT was 101.4 ± 2.8 s (mean ± SEM), 167.0 ± 4.5 s, and 97.8 ± 2.1 s in the no heparin, prophylactic heparin, and control groups, respectively. Mean activated partial thromboplastin time (aPTT) was 20.7 ± 0.4 s (± SEM), 46.5 ± 3.0 s, and 20.5 ± 0.4 s in the no heparin, prophylactic heparin, and control groups, respectively. White blood cell (WBC) count increased in all groups, suggesting an inflammatory response, but there was no significant difference in the final WBC count between the control and either device group (*p* = 0.37, one‐way ANOVA). Similarly, platelet count decreased and prothrombin time increased, indicating consumption of platelets and clotting factors, but there was again no difference between groups (platelets: *p* = 0.26; prothrombin: *p* = 0.99, one‐way ANOVA). For hemolysis, none of the groups had a statistically significant change in plasma hemoglobin versus baseline levels. Additionally, hematocrit increased slightly in all groups, suggesting no significant blood loss due to hemolysis or hemorrhage. The increase in hematocrit is likely a result of hemoconcentration secondary to mild dehydration (no maintenance fluids were given over the 6 h experiment).

## 3 DISCUSSION

In this study, we built a microfluidic oxygenator designed to support periviable infants in an artificial placenta circuit with minimal anticoagulation. We built and tested an initial prototype (Generation 1 Device), which provided performance data to inform design improvements. Our advanced prototype (Generation 2 Device) featured a smaller channel height, increased sweep gas pressure, and a modular multilayer design, which combined to significantly improve oxygen transfer. Using CFD, we evaluated features such as flow uniformity and wall shear stress. Finally, we tested the performance and hemocompatibility of the oxygenator using a neonatal pig model.

Over the past decade, microfluidic oxygenators have emerged as an alternative to hollow fiber membranes.^37^, ^38^, ^39^, ^40^, ^41^, ^42^ Microfluidic oxygenators contain microchannels, which can dramatically improve gas transfer and reduce priming volume.^43^ Typical microfluidic oxygenators are comprised of a flexible gas‐permeable elastomer. Despite several benefits over hollow fiber membranes, flexible microfluidic oxygenators are limited by frequent occlusion of the microchannels, which leads to high anticoagulation needs and large pressure drops.^10^ The silicon membrane oxygenator attempts to preserve the benefits of flexible microfluidics over hollow fiber membranes while overcoming the challenges associated with flexible microfluidic oxygenators. The key innovation is the rigid support structure of the semiconductor silicon backbone, which enables a flat plate design with short and wide channels. The short channel height enables high gas exchange efficiency, while the large width reduces flow resistance and the likelihood of occlusion. Additionally, for a given volume of blood, the use of wide channels allows for reduction of the area of the sidewalls, which are blood‐contacting surfaces that do not contribute to gas exchange. Finally, the flat plate nature of the membranes allows for precision control of the blood flow, potentially improving hemocompatibility.

The oxygen transfer exchange performance of the silicon membrane oxygenator is on par with that of recently published flexible microfluidic devices and hollow fiber membrane oxygenators. Our Generation 2 Device had a surface area normalized flux of 63 mL/min/m^2^ at a blood flow rate of 80 mL/min. A hollow fiber membrane oxygenator designed for an artificial placenta reported an efficiency of approximately 45 mL/min/m^2^ at 80 mL/min blood flow.^44^ In comparing to flexible microfluidic devices, it is challenging to directly compare the gas exchange performance of oxygenators designed to support different blood flow rates. However, recently published microfluidic devices report a surface area normalized flux of 31–164 mL/min/m^2^ for blood flow rates of 12–750 mL/min, suggesting that our device has a similar gas transfer efficiency.^40^, ^41^, ^42^ Carbon dioxide transfer was below our goal of 2.4 vol%. This was somewhat surprising, as carbon dioxide typically diffuses more readily than oxygen.^45^ The low carbon dioxide transfer may be due to carbon dioxide becoming trapped within “dead zones” in the pores or windows of the silicon membrane. Strategies to increase mixing on the sweep gas side of the membrane may help to improve carbon dioxide clearance.

The silicon membrane oxygenator had a minimal effect on markers of animal blood health. The rate of hemolysis, as measured by plasma free hemoglobin, was low. This is consistent with a low peak WSS of 3.0 Pa seen in our CFD analysis; hemolysis typically occurs at WSS >500 Pa.^31^ Although there was some degree of inflammation as well as consumption of platelets and coagulation factors, there was no difference between the device groups and the control group, suggesting that these changes may be due to the surgical cannulation process and placement on an extracorporeal circuit. Adding the silicon membrane oxygenator to the circuit did not significantly affect these markers of animal health.

The hemocompatibility profile of the silicon membrane oxygenator is encouraging. We tested our devices with no anticoagulation and with a prophylactic dose of unfractionated heparin. For the prophylactic dose heparin group, our target ACT level was 120–180 s. For comparison, a normal ACT in the absence of anticoagulation is 70–120 s. In pediatric ECLS with hollow fiber membrane oxygenators, a typical ACT target is 180–200 s.^46^ Additionally, in the clinical setting, oxygenators are typically coated with hemocompatible materials and run at a higher blood flow rate, both of which reduce the likelihood of thrombosis. For uncoated microfluidic oxygenators, ACT targets are even higher. Isenberg et al. targeted an ACT range of 250–350 s and reported a mean thrombosis percentage of 13.1% after 24 h of operation.^40^ We found a thrombosis percentage of 14.4% and 3.1% in the non‐anticoagulated and prophylactic anticoagulation groups, respectively, though our operation time was shorter (6 h). Although experimental variation makes it difficult to draw conclusive comparisons between our device and other microfluidics, our device is the only microfluidic oxygenator to be tested with low‐dose or no anticoagulation. A notable limitation of our experimental design was an inability to continuously measure pressure drop over time, a strategy frequently used as a surrogate for thrombus burden in oxygenator testing. Initial in vivo experiments included pre‐/post‐device pressure monitoring. However, in circuits with low blood flow rates (10 mL/min) and no therapeutic anticoagulation, we found that connections to the pressure monitors—sites of flow disruption and stasis—rapidly clotted within an hour. Future work with larger scale devices and higher circuit flow rates will likely enable continuous pressure monitoring.

This study identified several limitations of our current device design but also many potential improvements, which will be the focus of future work. Although our device oxygen transfer normalized to surface area was on par with other devices, we did not achieve our overall goal oxygen flux (3 vol%). Simply increasing the surface area for gas exchange could certainly allow us to achieve our goal flux. However, several other viable strategies were identified by this work. We found that decreasing channel height or increasing sweep gas pressure can improve gas transfer efficiency. Our Generation 2 Device had a channel height of 500 μm, which was an improvement over that of the Generation 1 device. However, it is still relatively large compared to microfluidics and hollow fiber membranes. The value of 500 μm was chosen as the shortest channel we could reproducibly fabricate with the available 3D printing technology. However, with advancements in 3D printing or use of precision machining, it should be possible to fabricate housings with shorter channel heights and thus improved efficiency. Another potential area of improvement is in the sealing of the membranes into the housing to allow higher sweep gas pressures. The rigid backbone of the silicon membranes can allow high sweep gas pressures (up to 50 PSI)^21^ without leaking or significant membrane deflection, a notable limitation of flexible microfluidic membranes.^40^ However, we found in our testing that at pressures higher than 15 PSI, the epoxy seal between the membranes and housing would occasionally reveal small leaks, resulting in gas bubbles within the blood channel. Improving the hermetic seal would enable reliable operation at higher sweep gas pressures and thus improved efficiency. Another consideration with regard to elevated sweep gas pressure is the possibility of gas emboli developing due to the local oxygen content exceeding the maximum carrying capacity of the blood. In our testing, the relatively low oxygen saturation of the blood (~70–90%) enabled the blood to fully absorb the pressurized oxygen. However, with future design iterations, if the outlet oxygen saturation is closer to 100%, the possibility of gas emboli formation will need to be considered. With regard to hemocompatibility, our data suggest that changes in the design of the blood flow path will reduce thrombosis. Based on our CFD analysis and in vivo testing, the inlet was prone to thrombosis due to stasis. For this work, we opted to design our prototypes to interface with an off‐the‐shelf tubing manifold, which necessitated a low‐shear transitional inlet. The results of this study suggest that redesigning the inlet and manifold to avoid low shear may further improve hemocompatibility. Additionally, redesigning the manifold using CFD could also help ensure equal flow distribution to the layers, a potential design limitation that was not assessed in this study. After the inlet/outlet, the next most common areas of thrombosis were the curved connections between the membranes, where flow dynamics were disrupted. Future designs will eliminate these curves in favor of straight channels. Finally, incorporation of hemocompatible coatings is likely to further reduce thrombosis. In the silicon membrane oxygenator, the material that contacts the blood is PDMS, which has excellent gas permeability but is not necessarily optimal for hemocompatibility. Many surface coatings have been developed for blood‐contacting medical devices,^47^, ^48^ and future work will evaluate these surface coatings for the silicon membrane oxygenator.

## 4 CONCLUSION

This work represents an important advance toward building an artificial placenta oxygenator designed to support periviable infants with minimal anticoagulation. The need for anticoagulation has been a critical barrier to the successful translation of an artificial placenta to humans. In this study, we demonstrate that a rigid and flat plate semipermeable membrane may offer a strategy to optimize the blood flow dynamics for hemocompatibility. We demonstrate that the silicon membrane oxygenator can be scaled up using a multilayered modular design. Additionally, this device is the only microfluidic oxygenator that has been shown to operate with subtherapeutic anticoagulation. Our work identified 4 key areas for further improvement: (1) reducing the channel height further, (2) improving sealing to allow higher pressure sweep gas, (3) refinement of the blood flow path, especially the inlet, manifold, and curved sections, and (4) addition of hemocompatible coatings. By combining this future work with the findings of this study, the silicon membrane oxygenator has the potential to realize the goal of an anticoagulation‐free artificial placenta.

## 5 MATERIALS AND METHODS

### 5.1 Silicon membrane fabrication

Silicon on insulator (SOI) wafers were purchased from MEMS Engineering & Material, Inc. (Sunnyvale, CA, USA). They contained a “device” layer of 100 μm, a buried silicon dioxide (“oxide”) layer of 1 μm, and a “handle” layer of 299 μm for an overall thickness of 400 μm. A layer of photoresist (SPR‐220, 3 μm thick) was spin coated onto the device layer of the wafer and patterned with pores 10 μm wide by 50 μm long, separated by 10 μm on all sides. The device layer was etched with deep reactive ion etching (DRIE) to the buried oxide layer. On the handle layer, the process was repeated except with larger 1 mm by 6 mm by 299 μm windows (width × length × depth). The membrane design resulted in an active gas exchange area (i.e., window area) of 37% and 61%, relative to the total and blood‐contacting surface area, respectively. The corresponding pore areas were 15% and 25%. Following etching, the wafers were diced into 32 mm × 65 mm membranes. After dicing, the membranes were wet etched using 49% hydrofluoric acid, which dissolved the 1 μm buried oxide layer, thereby connecting the pores with the windows and completing the semiconductor silicon membrane backbone.

To build the 5 μm layer of PDMS, first an initial sacrificial layer of approximately 250 μm PDMS (Sylgard 184, Dow Corning, Midland, MI, USA) was spin coated onto a silicon wafer (10:1 base to crosslinker mix ratio, 500 RPM for 20 s, cure at 80°C for 2 h). After curing, the surface was treated with oxygen plasma (Expanded Plasma Cleaner, Harrick, Ithaca, NY, USA) at 30 watts for 20 s to increase surface wettability. A second sacrificial layer (approximately 2–5 μm) of polyvinyl alcohol (PVA; Sigma‐Aldrich #P8136, St. Louis, MO; powder diluted 5% w/w in water) was spin coated on top of the PDMS layer (1000 RPM for 60 s, cure at 60°C for 1 h). Finally, the 5 μm layer of PDMS was created by diluting a 10:1 mixture of PDMS with an equal amount of hexane (Sigma‐Aldrich #296090, St. Louis, MO) and spin coating the PDMS/hexane mixture onto the PVA layer (5000 RPM for 300 s, cure at 80°C for 2 h).

The final composite membrane was created by combining the silicon membrane backbone and the thin PDMS layer. The PDMS construct was peeled off the silicon wafer and treated with oxygen plasma along with the silicon backbone (30 W for 20 s). The PDMS construct was then wetted with distilled water, and the 5 μm side was placed in contact with the pores side of the semiconductor silicon backbone, taking care to eliminate air bubbles. The pieces were bonded together under moderate weight on a hotplate for 12 h at 70°C. After bonding was complete, the excess PDMS beyond the borders of the silicon chip was cut away. The composite membrane was submerged in distilled water at 70°C for 4 h to dissolve the PVA and release the sacrificial PDMS layer, leaving the completed composite membrane.

Prior to use in the oxygenator, all membranes were individually tested for defects using a custom flow cell. Water was pumped into a chamber on the front “PDMS” side, and compressed air was pressurized on the back side up to a total of 20 PSI. The location of any bubbling was noted as a defect (typically 1–3 per membrane) and covered with epoxy (Epo‐Tek OD2002, Epoxy Technology, Billerica, MA, USA) on the window side. This process was repeated until the membranes were able to withstand 20 PSI with no bubbling for at least 1 min.

### 5.2 First generation device construction

An eight‐membrane stack was created by bonding individual composite membranes to molded gasket spacers. The gaskets were created using PDMS (Sylgard 184, 10:1 mix ratio) poured into custom‐machined aluminum molds. Blood channel gasket dimensions were 2.5 mm by 65 mm by 2 mm (width × length × height)—the 2 mm gasket height corresponds to the blood channel height. Sweep gas gaskets were all 700 μm in height. Edge gaskets were L‐shaped and 32 mm on the short arm and 57 mm on the long arm to allow a 4 mm opening on the sides for the sweep gas inlet and outlet. Additional gaskets of 3 mm width and 50 mm length were placed in the middle for structural support. The gaskets were all bonded to a single side of a chip (blood gaskets to the front side and sweep gas gaskets to the back side) using PDMS crosslinker.^29^ Prior to bonding, all gaskets were measured using a micrometer, and only included if the dimensions were within 1% of the target measurement. The crosslinker was spin coated (1000 rpm for 60 s) on a silicon wafer to form a thin layer of approximately 3–5 μm. The gaskets were then “stamped” onto the crosslinker and aligned using a custom‐machined alignment rig with the uncured crosslinker face up. The silicon membranes were lightly pressed onto the gaskets. Finally, the silicon membrane with aligned gaskets was placed onto a hotplate and cured under moderate weight (80°C for 4 h). Once gaskets were bonded to a single side, the stamping process was repeated on the other side of the gaskets to form the full stack. The entire stack was composed of 8 silicon membranes flanked by 2 silicon solids (no pores or PDMS).

Once the stack was constructed, it was placed into a 3D printed (Stratasys J750 PolyJet printer, VeroClear) housing by sliding the stack along rails printed in the housing, which ensured proper interfacing of the blood channels in the housing and the stack. Sweep gas headers were attached to either side of the stack to connect the main sweep gas inlet and outlet of the housing to the 4 mm openings between the L‐shaped gaskets in the sweep gas channels of the stack. The stack was then sealed on all edges with medical epoxy (Masterbond EP30MED, Hackensack, NJ, USA). Two side plates and a top plate were screwed in place for additional mechanical support.

### 5.3 Second generation device construction

For the Generation 2 Device, the design was altered to utilize two‐membrane stacks with blood channel gaskets. Gaskets were molded from PDMS and had dimensions of 2 mm by 65 mm by 500 μm (width × length × height), thus defining a blood channel height of 500 μm. The gaskets were bonded to the membranes using a custom alignment rig and PDMS crosslinker, as described above. Each layer consisted of 5 stacks (10 membranes total). The 5 stacks were placed into a 3D printed housing (PolyJet, Veroclear) using a shelf that aligned the membrane channels with connecting channels in the housing, thus creating a continuous blood flow path. The stacks were then sealed within the housing using epoxy. The multilayered device was assembled by stacking the layers with a 1 mm silicone gasket separating each layer. The silicone gasket was designed to allow sweep gas to flow between the layers. Finally, a tubing manifold was connected to distribute the blood to each layer in a parallel flow manner.

### 5.4 Benchtop flow cell testing

Membranes were tested at various channel heights and sweep gas pressures using a benchtop mock circuit loop. A water reservoir was filled with distilled water and sparged with nitrogen for 2 min to achieve a partial pressure of oxygen between 50 and 100 mmHg. The water was then pumped using a peristaltic pump (Masterflex L/S Series, Cole‐Parmer, Vernon Hills, IL, USA) through the device at a rate of 10 to 40 mL/min. The oxygen concentration was measured in μmol/L using an optical oxygen sensing probe (NeoFox, Ocean Insight, Orlando, FL, USA). After measuring the baseline oxygen concentration, the oxygen sweep gas was turned on and pressurized to up to 10 PSI. The oxygen concentration was allowed to stabilize over 1 minute, and the value recorded. Oxygen flux (J) at standard temperature and pressure (STP) was calculated using Equation (1), where *q*_w_ is the volumetric flow rate of water (mL/min), *R* is the ideal gas constant, and *n*_f_ and *n*_i_ are the respective final and initial molar concentrations of oxygen (μmol/L) measured by the oxygen probe.

(1) $$J=q_{w}R\left(n_{f}-n_{i}\right)$$

### 5.5 Mass transfer and computational fluid dynamics modeling

Computational fluid dynamics modeling work was performed using Ansys software (Ansys, Canonsburg, PA, USA). The blood flow path was built in Solidworks (Dassault Systèmes, Vélizy‐Villacoublay, France), imported into Ansys Meshing, and converted into a mesh containing ~40,000 hexahedral‐shaped elements. Ansys Fluent was used to conduct the laminar flow CFD modeling. A density of 1060 kg/m^3^ and a Cross non‐Newtonian viscosity model were used to define blood properties.^26^, ^27^

Oxygen flux modeling was performed using a mass transfer model coded in MATLAB (MathWorks, Natick, MA, USA). The mathematical derivation of the oxygen mass transfer model is described in detail in Dharia et al.,^17^ and custom MATLAB files are available for download in Supplementary Material. The modeling constants used for both the CFD and mass transfer models are summarized in (Table S5, Supplementary Material) and are based on known properties of fetal blood.^28^

### 5.6 Extracorporeal gas exchange testing

Testing of the oxygenator was performed in an extracorporeal pig model at Covance Lab (San Carlos, CA, USA). All procedures were approved by the Institutional Animal Care and Use Committee at Covance (ANS2764). To minimize animal numbers, the pigs used for gas exchange testing were part of a separate study evaluating an implanted hemodialysis device. Oxygenator gas exchange testing was performed following explant of the hemodialysis device prior to euthanasia. Adult pigs (~70 kg, mixed male/female) were anesthetized and endotracheally intubated. Intraoperative monitoring included continuous pulse oximetry, arterial blood pressure measurements, and cardiorespiratory monitoring. A 12 French double lumen venous dialysis catheter (Mahurkar Elite #8888221213, Medtronic, Minneapolis, USA) was placed in the jugular vein and connected to the oxygenator. Pigs were anticoagulated with intravenous unfractionated heparin to achieve an activated clotting time 200–250 s. For single‐layer devices, blood was pumped at 10 mL/min using a peristaltic pump. For multilayered devices, blood was pumped at 10 mL/min/layer (i.e., up to 80 mL/min for an 8‐layer device). Pressure drop was monitored using pressure transducers (Edwards Lifesciences, Irvine, CA, USA) placed before and after the device. To measure oxygen flux, 100% oxygen sweep gas was turned on and pressurized up to a maximum of 10 PSI. The sweep gas was titrated based on pressure (as opposed to flow) but varied between 0.1 and 1 L per minute. Blood samples were taken from ports before and after the device, and blood gas analysis was performed using an i‐STAT blood gas analyzer (Abbott Point of Care Diagnostics, Princeton, NJ, USA). Volumetric oxygen content ([O_2_]) of the blood (mL/dL) was calculated using Equation (2), where Hgb is the hemoglobin concentration (g/dL), S_O2_ is the oxygen saturation, and P_O2_ is the partial pressure of dissolved oxygen (mmHg). Carbon dioxide ([${\mathrm{CO}}_{2}]$) content (mL/dL) was estimated using Equation (3), where P_CO2_ is the partial pressure of carbon dioxide (mmHg).^45^, ^49^ The oxygen or carbon dioxide flux was then determined by multiplying the change in content by the blood flow rate.

(2) $$\left[O_{2}\right]=1.36\times \mathrm{Hgb}\times S_{O2}+0.0031\times P_{O2}$$

(3) $$\left[{\mathrm{CO}}_{2}\right]=12.8105\times P_{\mathrm{CO}2}^{\;0.3692}$$

### 5.7 In vivo hemocompatibility testing

Hemocompatibility testing was performed using a neonatal pig model at Nemours Children's Health (Wilmington, DE). All procedures were approved by the Institutional Animal Care and Use Committee at Nemours (RSP23‐56599‐001). Neonatal pigs (~4 kg, mixed male/female) were anesthetized, endotracheally intubated, and underwent placement of 5 French single lumen catheters (Vygon #1270.05, Lansdale, PA, USA) in the jugular vein and carotid artery. A “mock” oxygenator device was then connected to the circuit. The mock device consisted of glass membranes coated with a 5 μm layer of PDMS. Glass was used for its transparency, which allowed visualization of clots. Despite the use of glass instead of silicon as the membrane “backbone,” the blood‐contacting side of the membrane remained PDMS. Blood was pumped through the mock oxygenator at 10 mL/min, and the device was visually monitored continuously for thrombus development. Photos of the device were taken with any new clot development and at least every 2 h. The photos were analyzed in MATLAB, and the area of thrombus was quantified by drawing regions of interest (ROIs) around all clots. The level of thrombosis was calculated by dividing the thrombus area by the total area of the blood path and expressed as a percentage. Additionally, blood samples (from an arterial line in the contralateral carotid artery) were collected at baseline and every 2 h until completion of the 6 h study. A complete blood count quantified white blood cell count, hematocrit, hemoglobin concentration, and platelet count using a Micros ESV60 Veterinary Hematology Analyzer (Horiba, Irvine, CA, USA). Activated clotting time, prothrombin time, and activated partial thromboplastin time were analyzed with a Hemochron Signature Elite (Werfen, Barcelona, Spain). Plasma free hemoglobin was measured by centrifuging whole blood at 2000 g for 15 min followed by measurement of plasma hemoglobin using the Hemocue Plasma/Low Hgb Analyzer (Hemocue, Angelholm, Sweden).

## AUTHOR CONTRIBUTIONS

Conceptualization: DGB and SR. Methodology: DGB, NCH, and SR. Investigation: DGB, NCH, AH, BD, NW, PN, JM, BWC, and FJB. Visualization: DGB. Funding acquisition: DGB and SR. Project administration: DGB, CB, and SR. Supervision: DGB and SR. Writing – original draft: DGB. Writing – review and editing: DGB, NCH, AH, BD, NW, PN, JM, BWC, CB, and SR.

## FUNDING INFORMATION

National Institutes of Health Grant T32 HD049303 (DGB). National Institutes of Health Grant T32 HL007544 (DGB). National Institutes of Health Grant U01 EB025136 (SR). National Institutes of Health Grant R21 HD113527 (SR, DGB). Department of Defense Grant PR210482 (SR). West Coast Consortium for Technology and Innovation in Pediatrics, Catalyzing Pediatric Innovation Grant (DGB). UCSF‐Stanford Pediatric Device Consortium, UCSF‐Benioff Hospitals Prize, 2020 (DGB). UCSF‐Stanford Pediatric Device Consortium, Hooper Family Prize, 2022 (DGB). Thrasher Early Career Award (DGB). Boston Children's Hospital Anesthesia Ignition Award (DGB).

## CONFLICT OF INTEREST STATEMENT

SR is the founding director of Silicon Kidney, LLC and an advisory board member for Vitara Biomedical. SR is a co‐inventor for the awarded patent, “Gas Exchange Composite Membranes and Methods of Use Thereof,” (#20240342352). The authors declare no other competing interests.

## DATA AND MATERIALS AVAILABILITY

The data that support the findings of this study are available from the corresponding author upon reasonable request.

## Supporting information


**DATA S1.** Supporting Information.
