# Supplementary material for: A silicon membrane microfluidic oxygenator for use as an artificial placenta with minimal anticoagulation
Source: Bioeng Transl Med. 2025 Jul 12;10(5):e70037. doi: 10.1002/btm2.70037 (PMC12478335; doi:10.1002/btm2.70037)
Supplement: Supplementary file 1 — DATA S1. Supporting Information. [file BTM2-10-e70037-s001.docx]

**Table S1:** Expected Patient Values and Target Design Parameters

| **Patient Parameter*** | **Value** |
| --- | --- |
| Weight | 330-570 g |
| Blood volume | 46-80 mL |
| Placental Flow | 200 mL/kg/min |
| Umbilical artery saturation | 50% |
| Umbilical vein saturation | 80% |
| Oxygen demand | 6 mL/kg/min |
| Umbilical artery pressure | 40-50 mmHg |
| Umbilical vein pressure | 5-10 mmHg |
| **Specification** | **Target Parameter** |
| Pressure drop | <30 mmHg |
| Flow Rate | 67-133 mL/min |
| Oxygen Flux | 2-3.5 mL/min (~3 vol%) |
| Wall Shear Stress | 1-3.6 Pascals |
| Anticoagulation | None or “Prophylactic Dose” |

Target patient = 20-23 week gestational age infant

Spin coat and

pattern photoresist

Deep reactive

ion etching

Clean off

photoresist

Deep reactive

ion etching

Clean off

photoresist

Wet etch with

hydrofluoric acid

a

b

Spin coat and pattern backside photoresist

Spin coat a sacrificial

PDMS layer

Spin coat a dissolvable

PVA layer

Spin coat the thin

PDMS layer

c

Oxygen plasma bond the thin PDMS layer to the silicon chip

Dissolve the PVA layer, releasing the sacrificial PDMS layer

Final composite

membrane


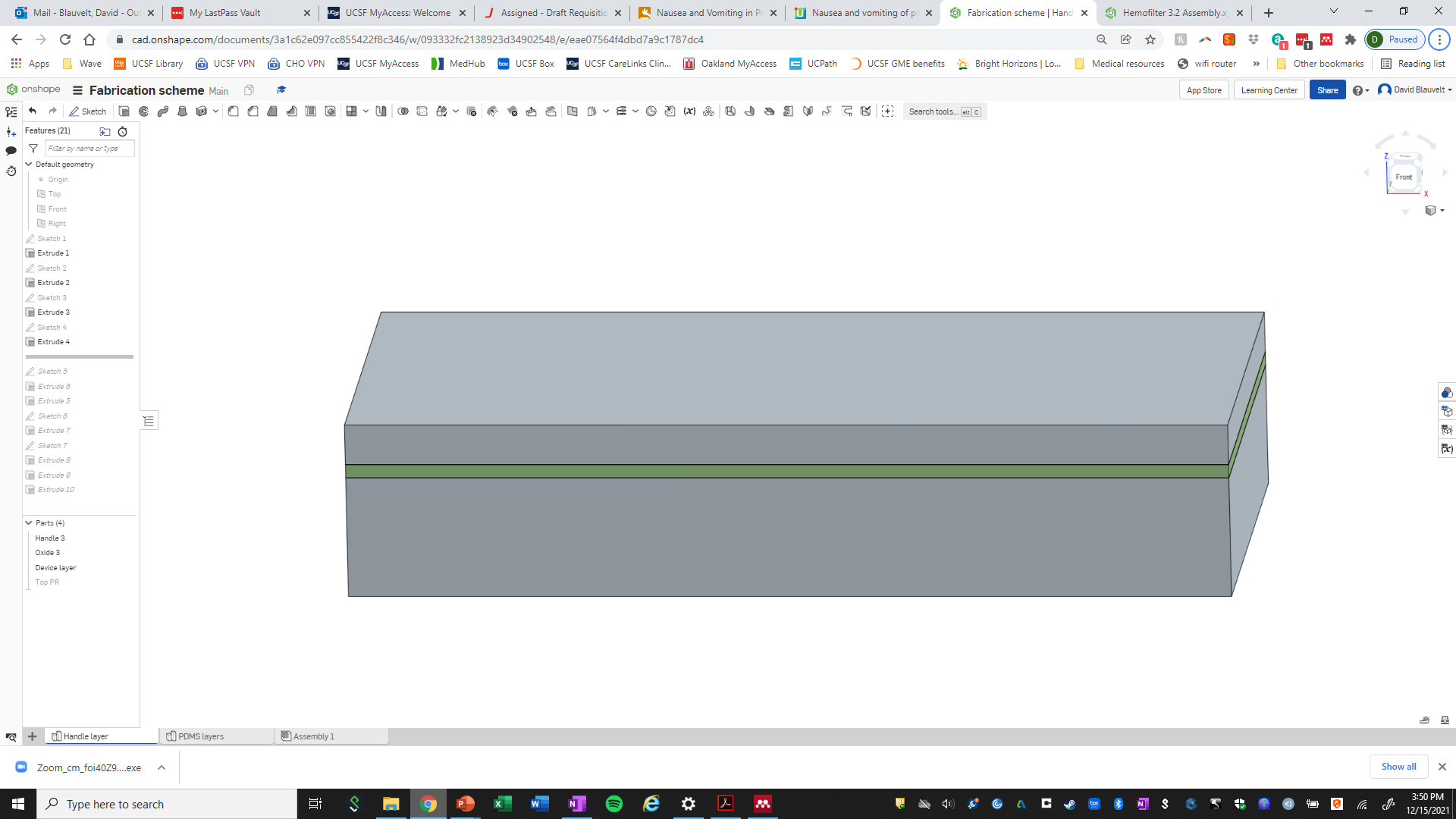

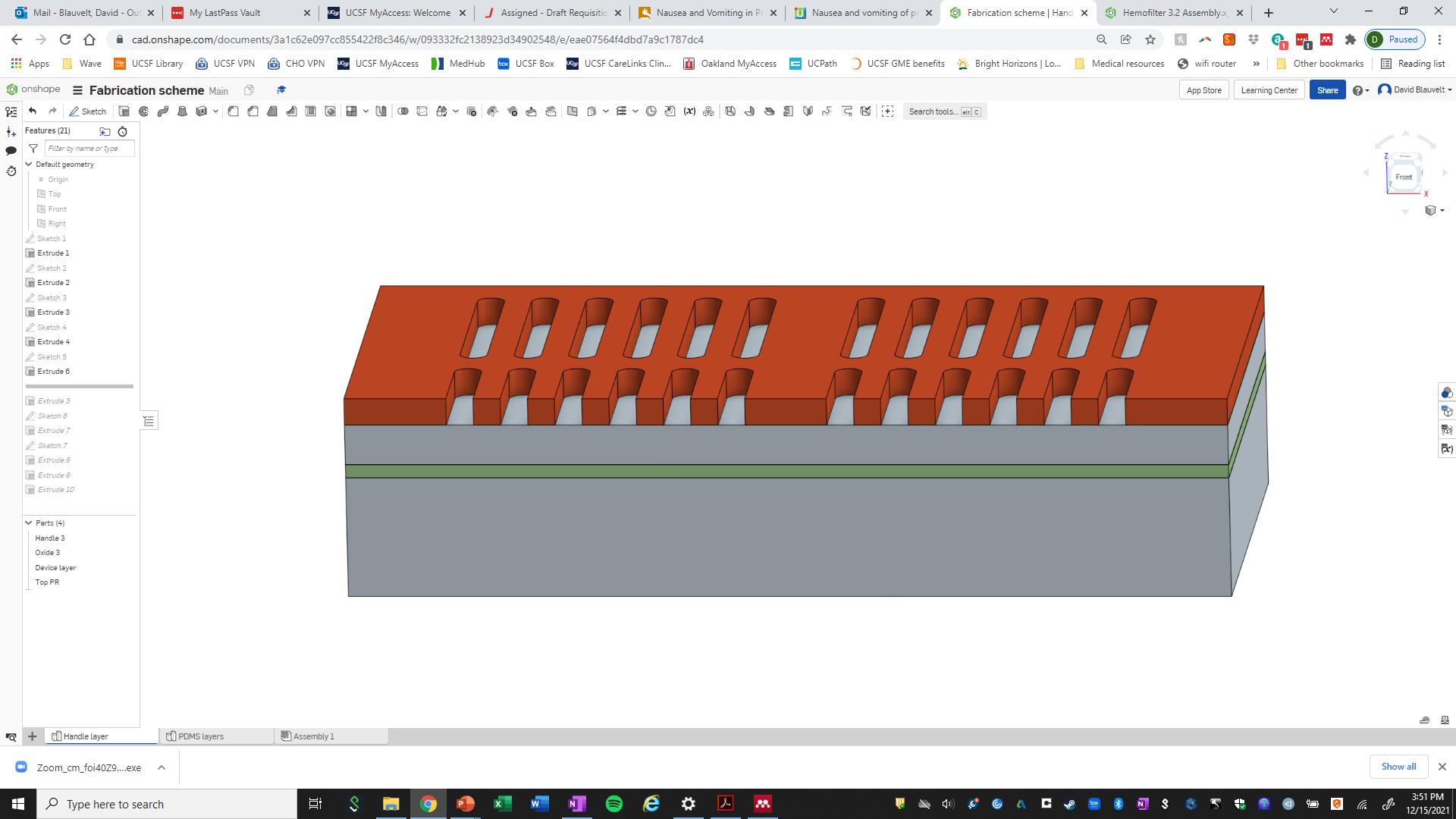

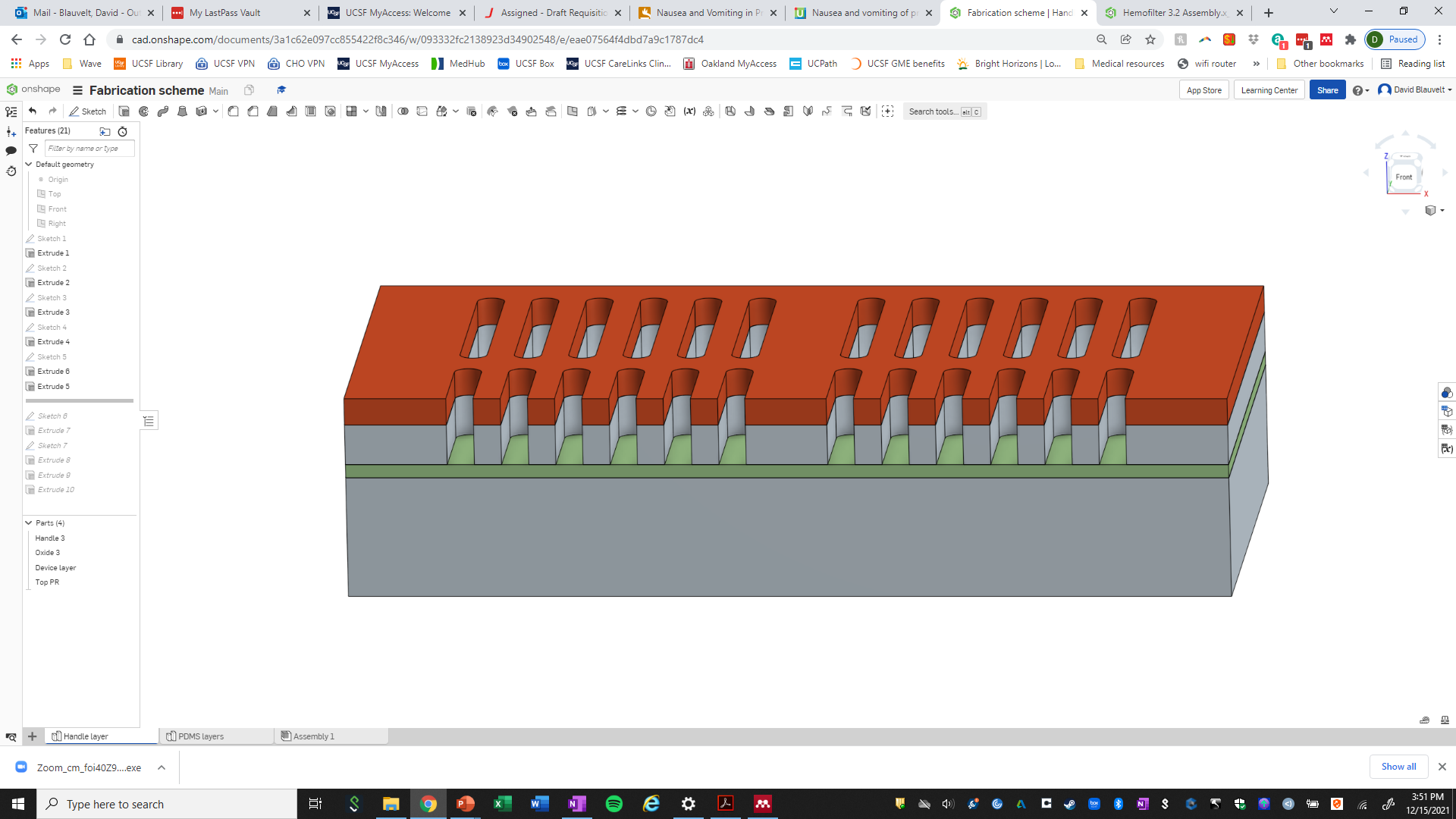

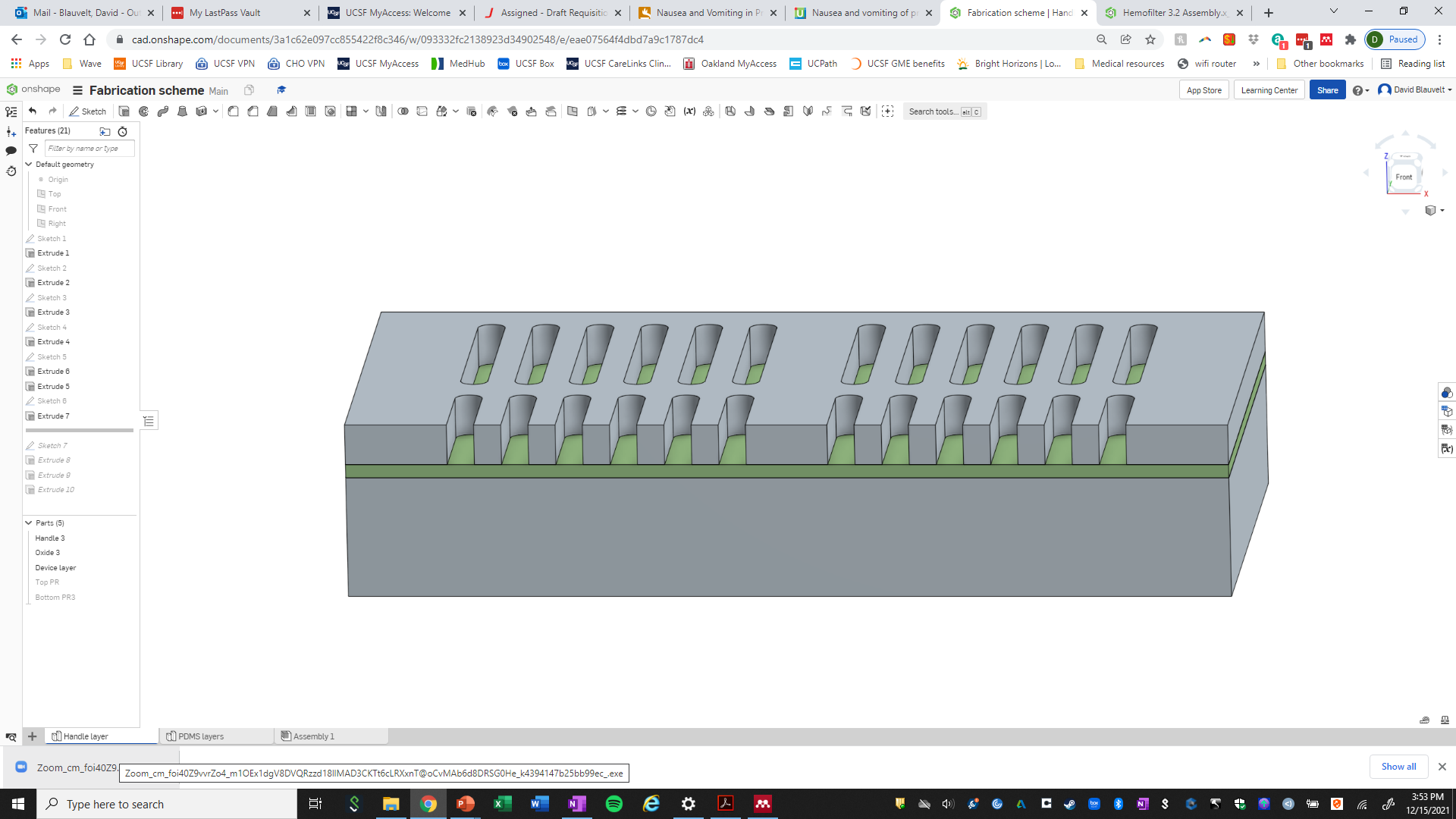

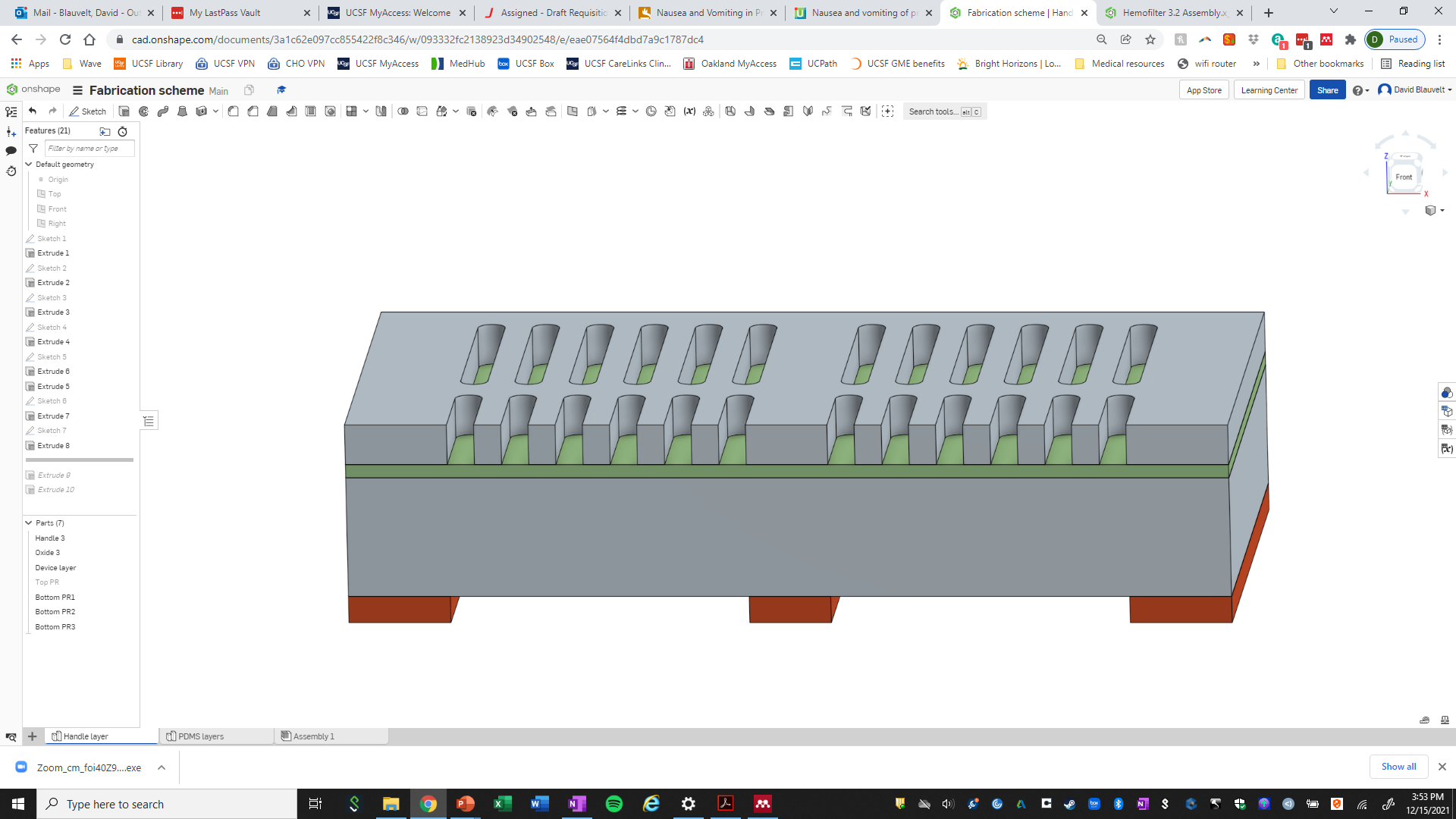

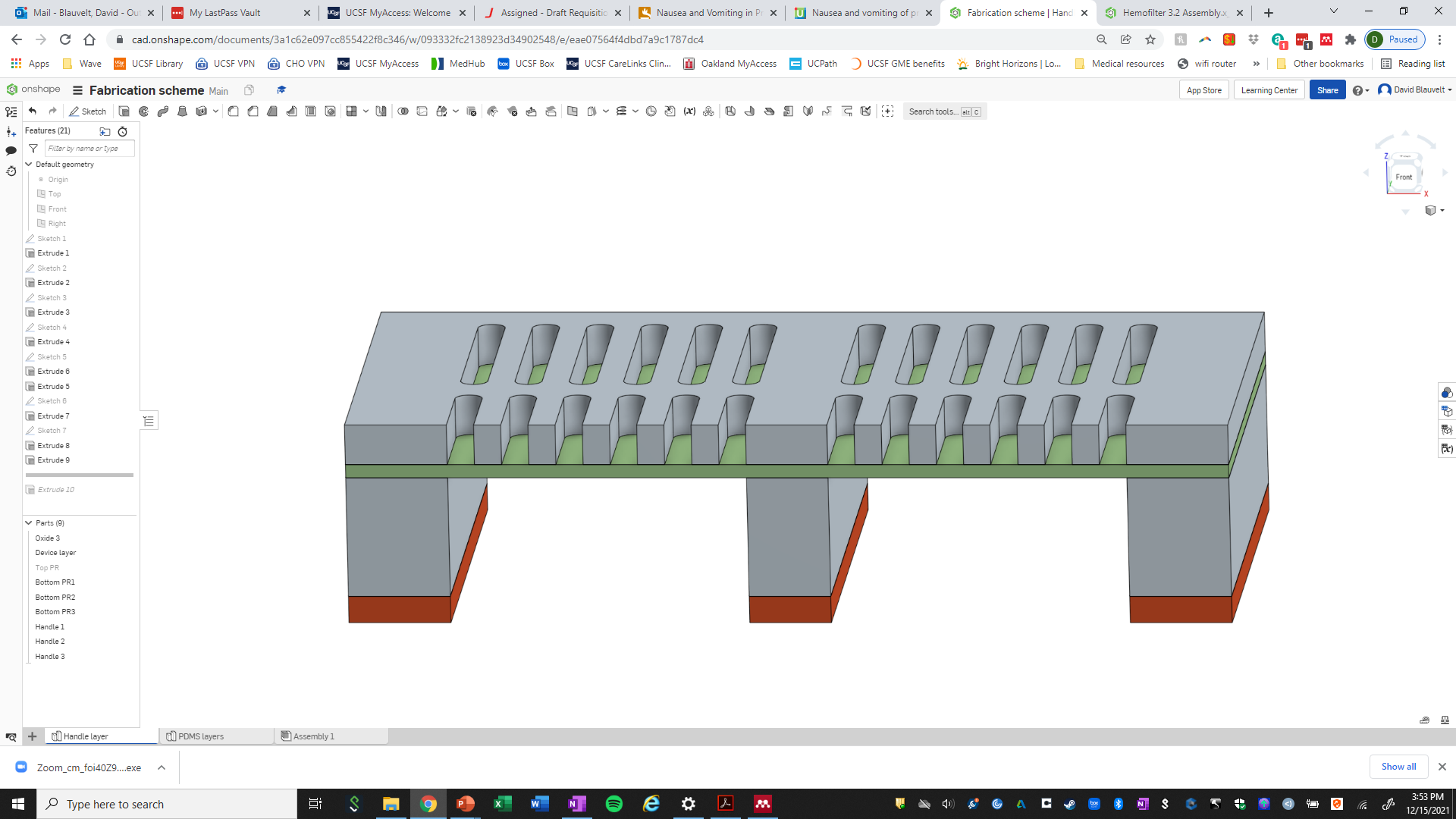

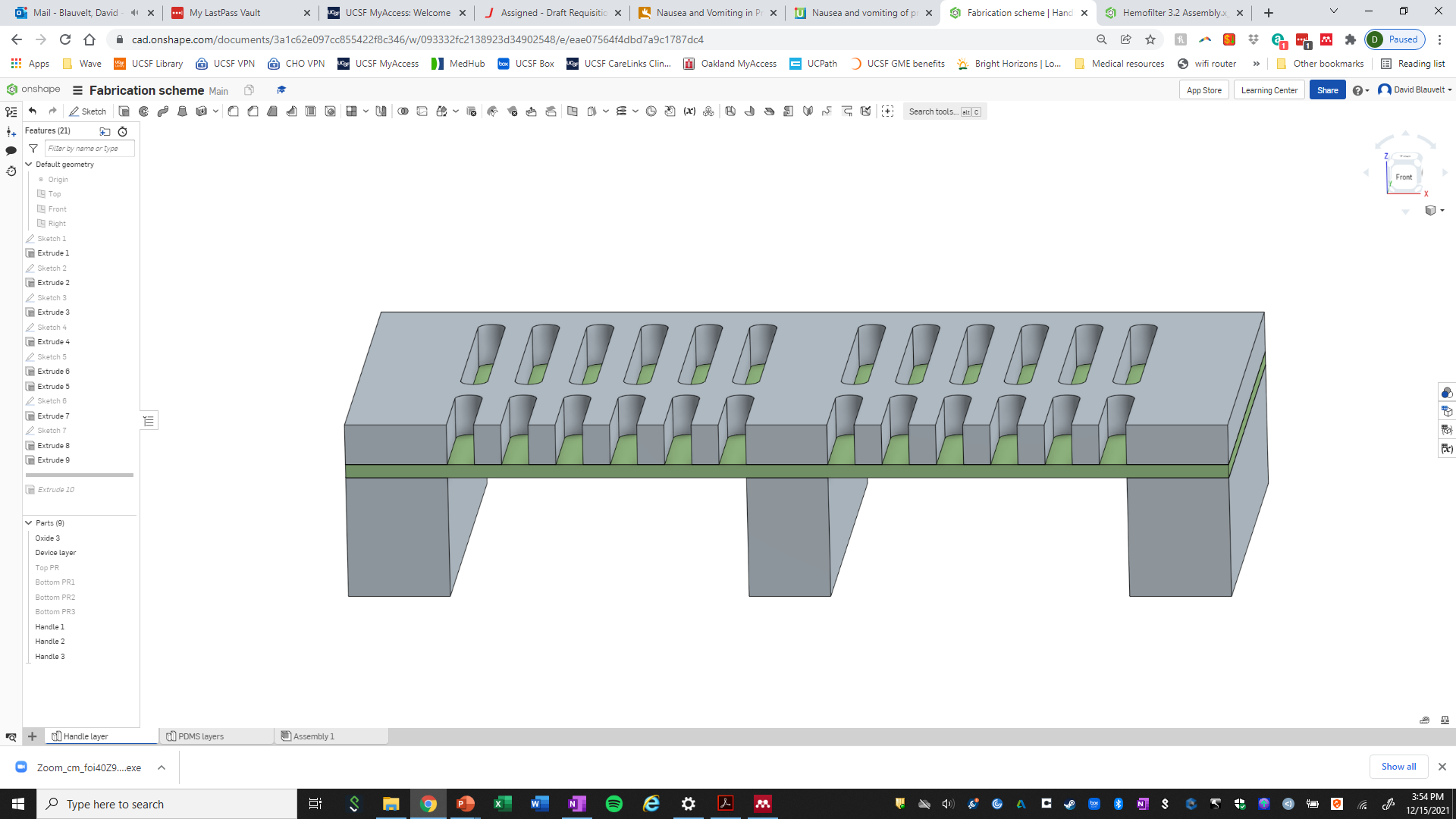

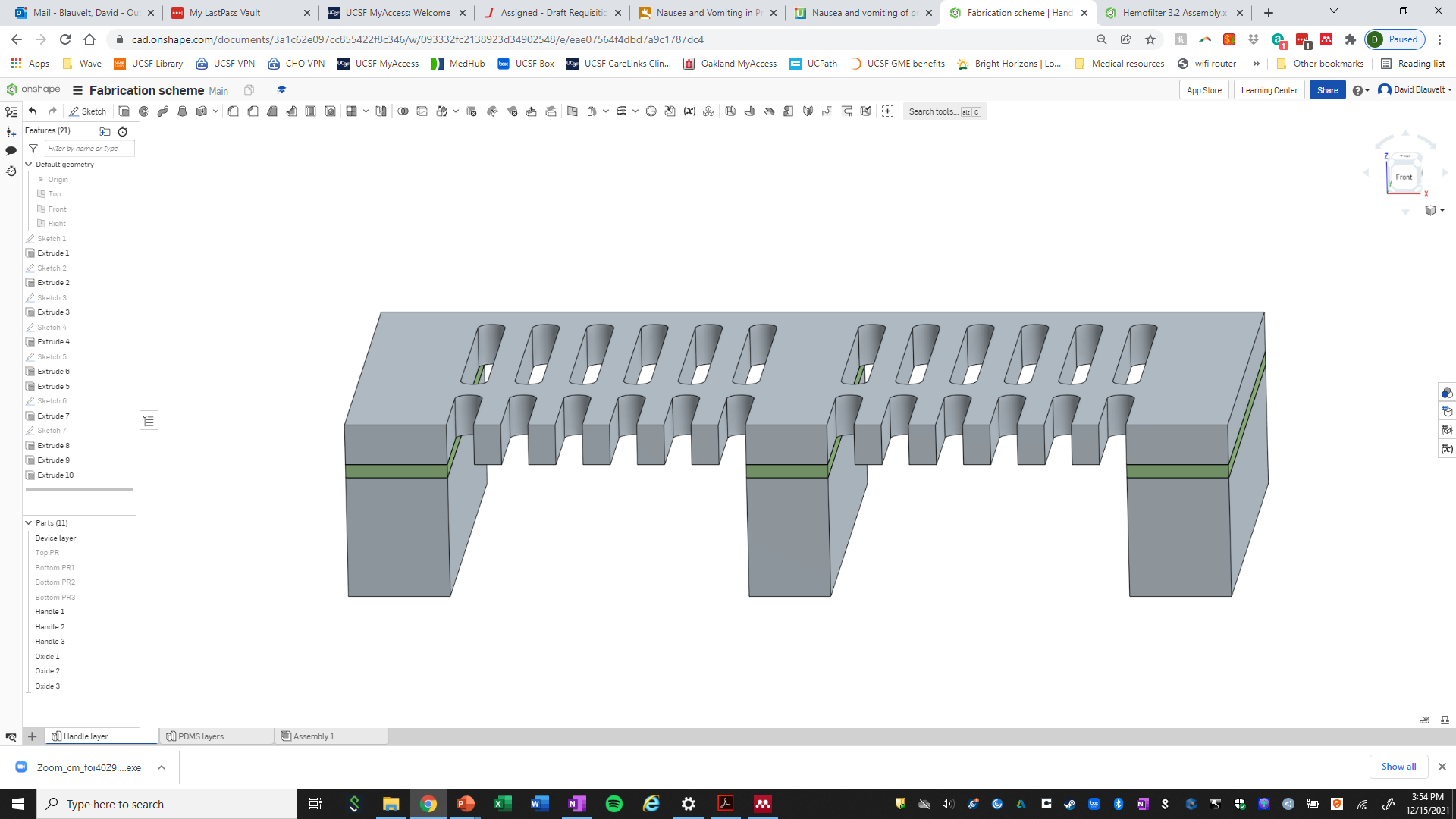

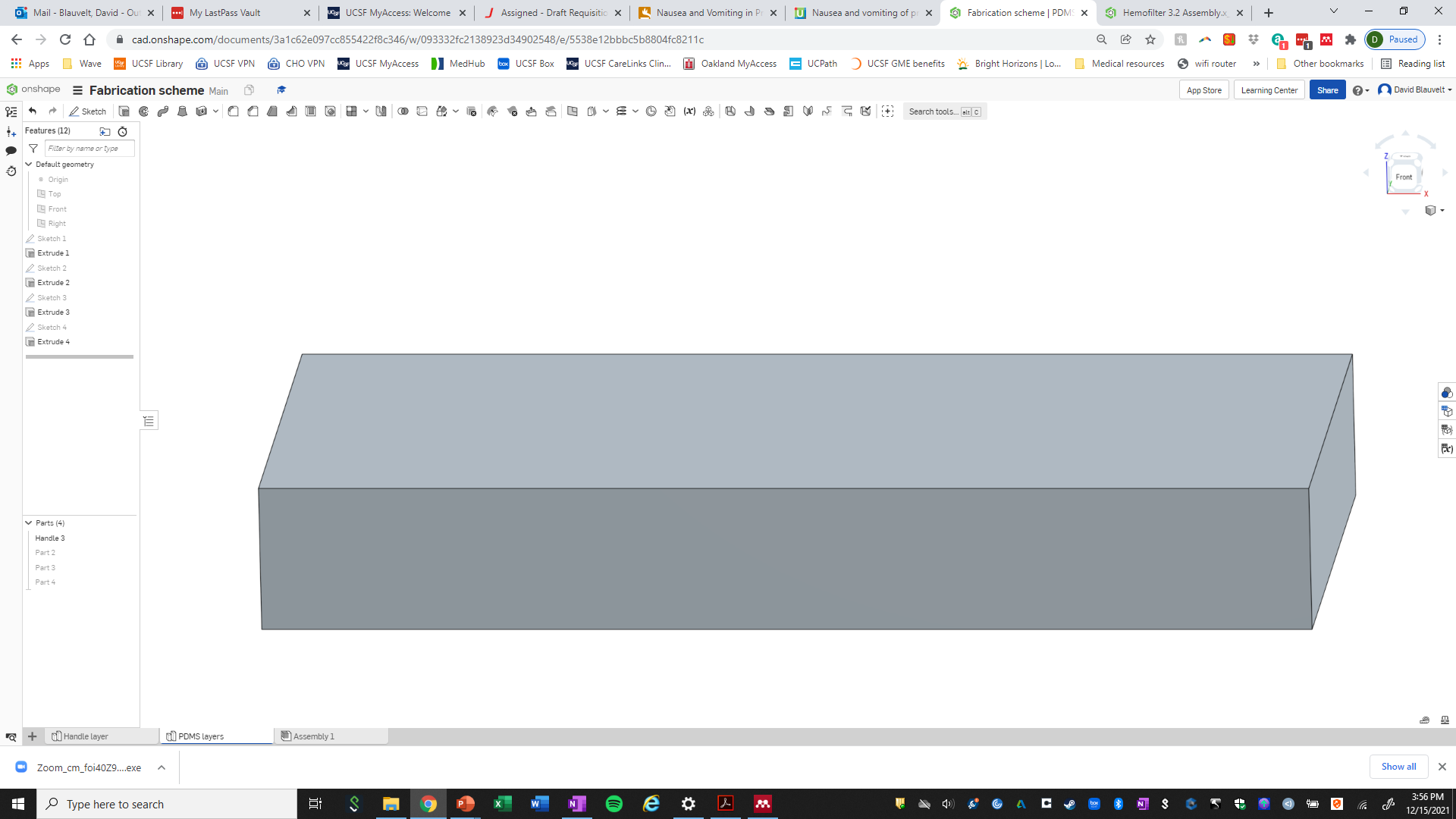

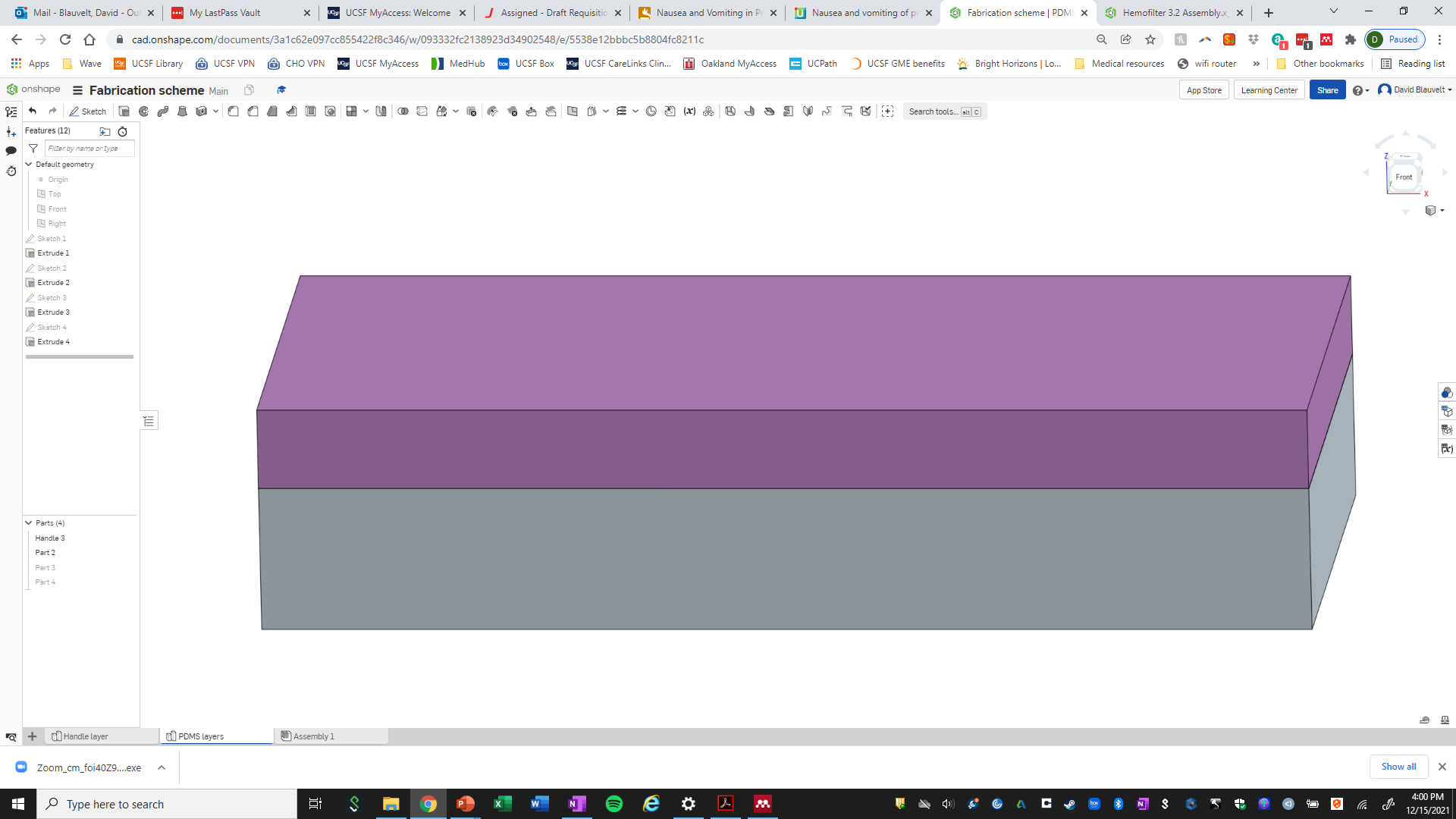

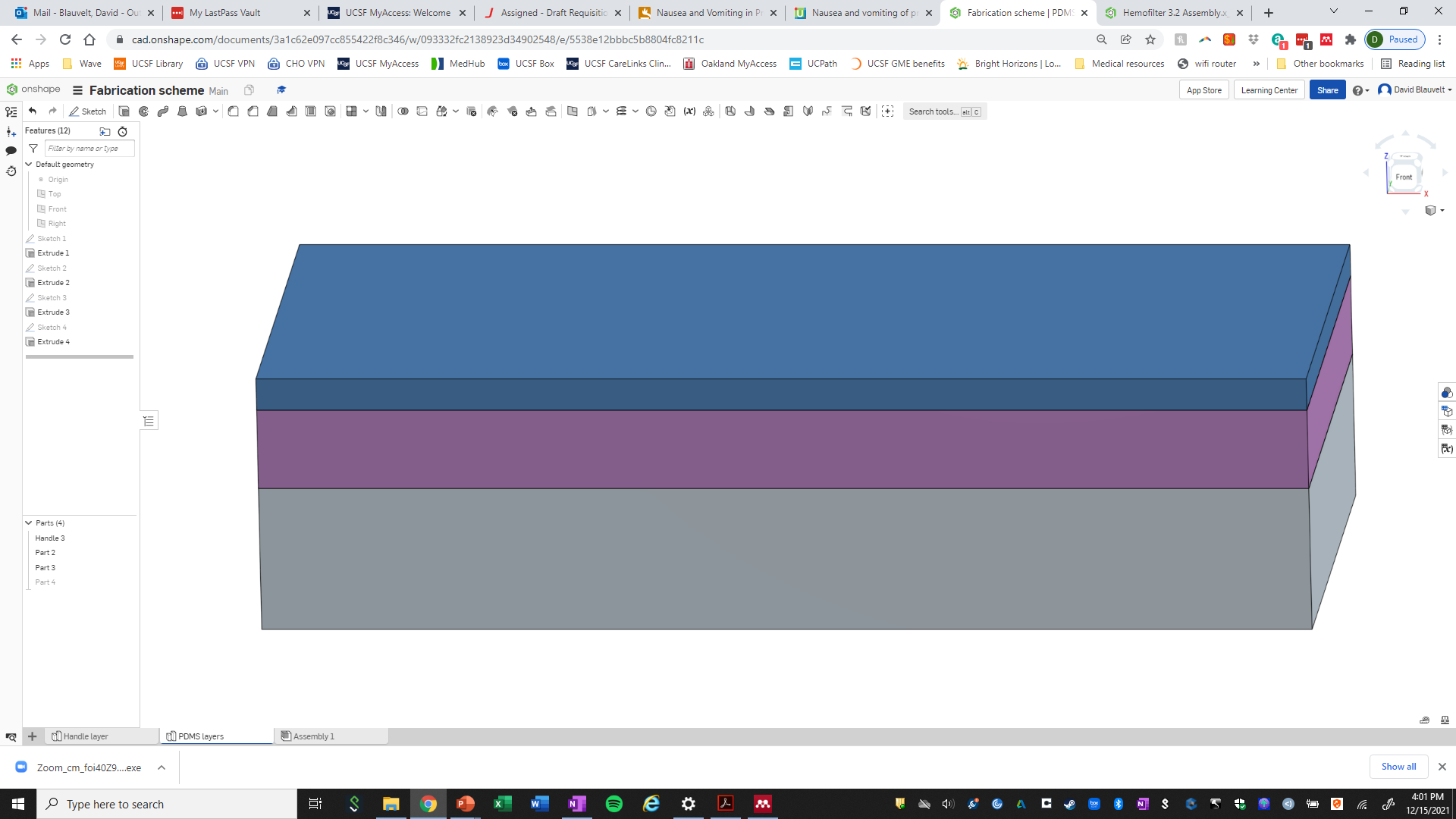

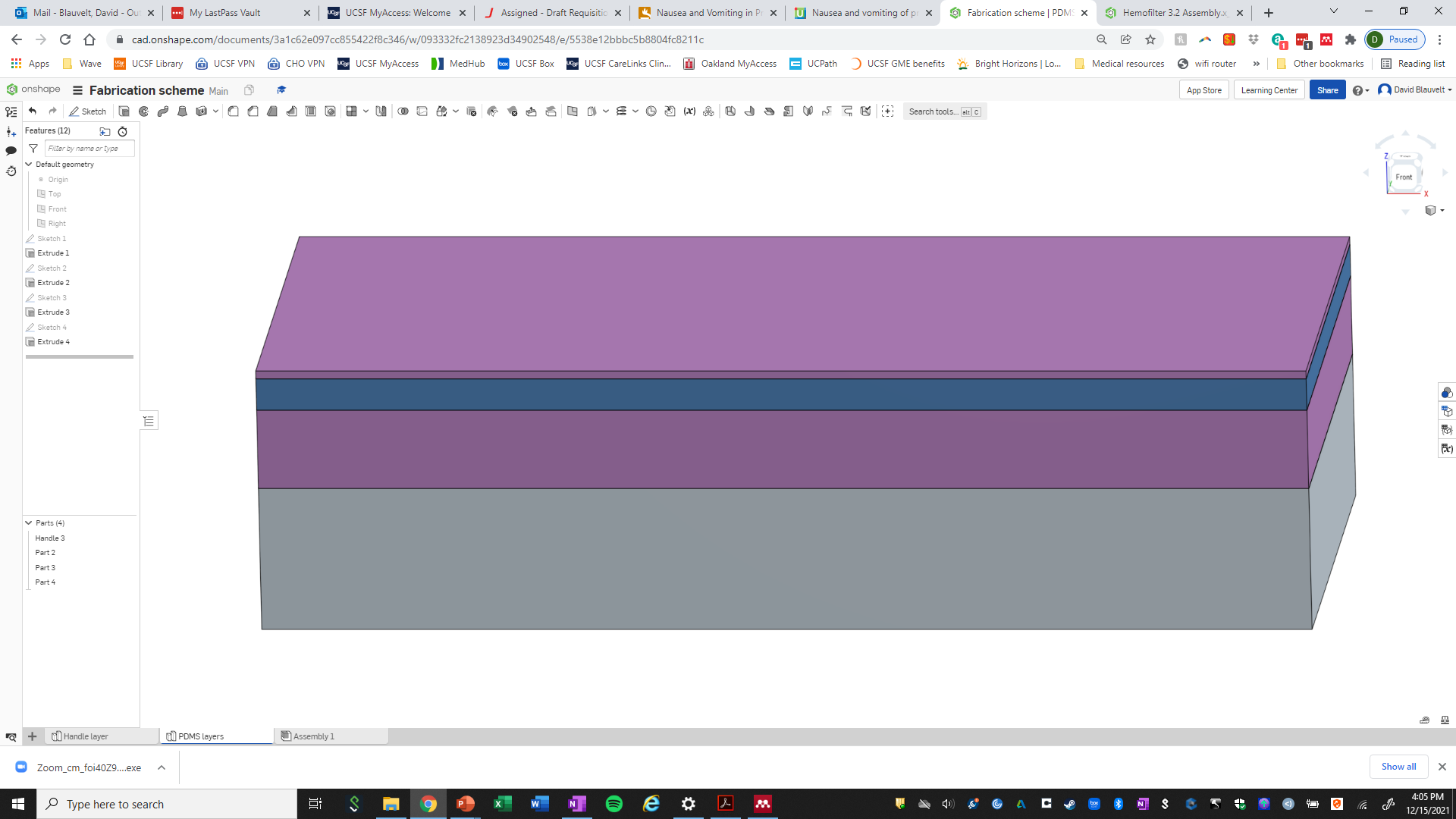

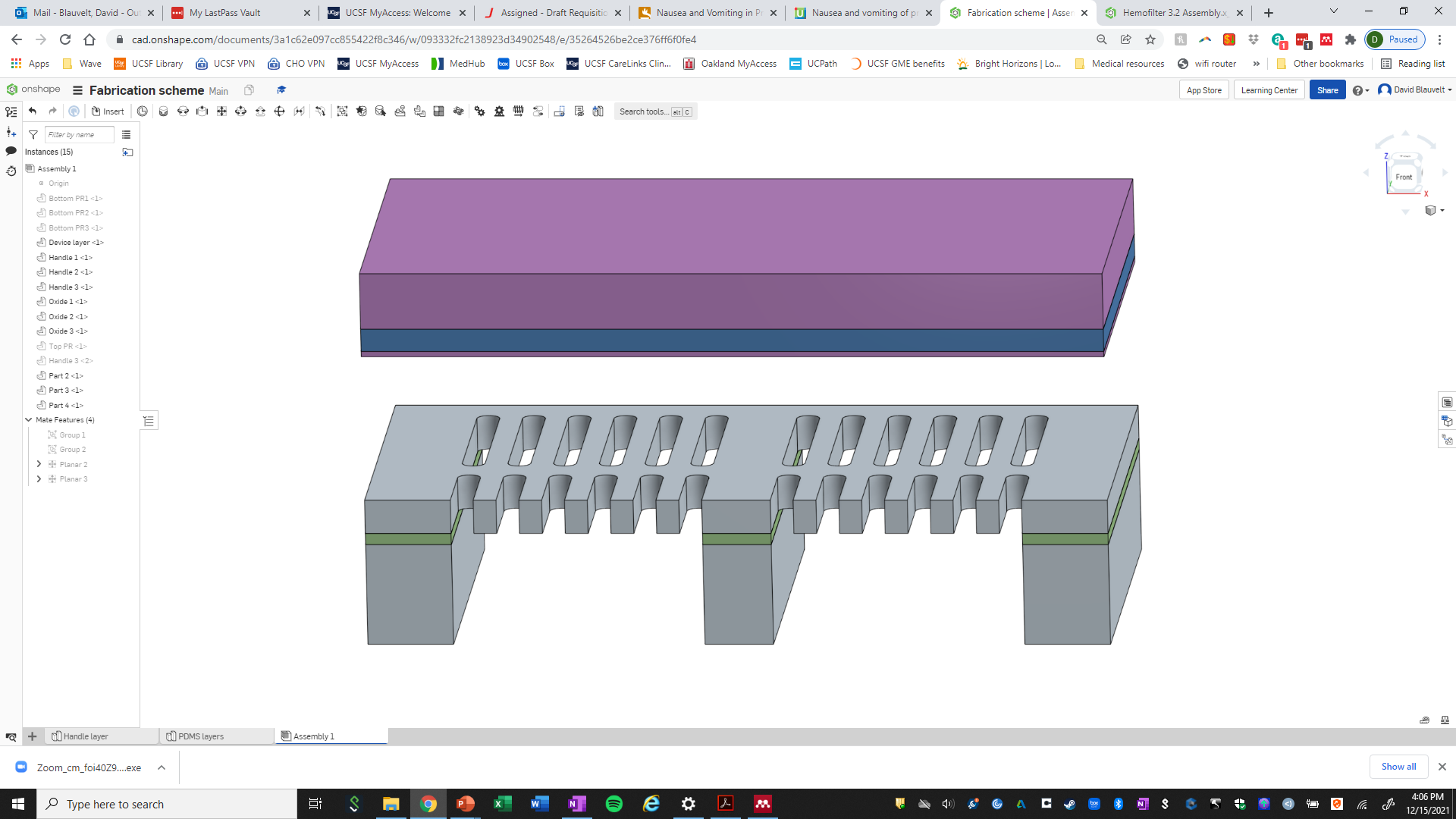

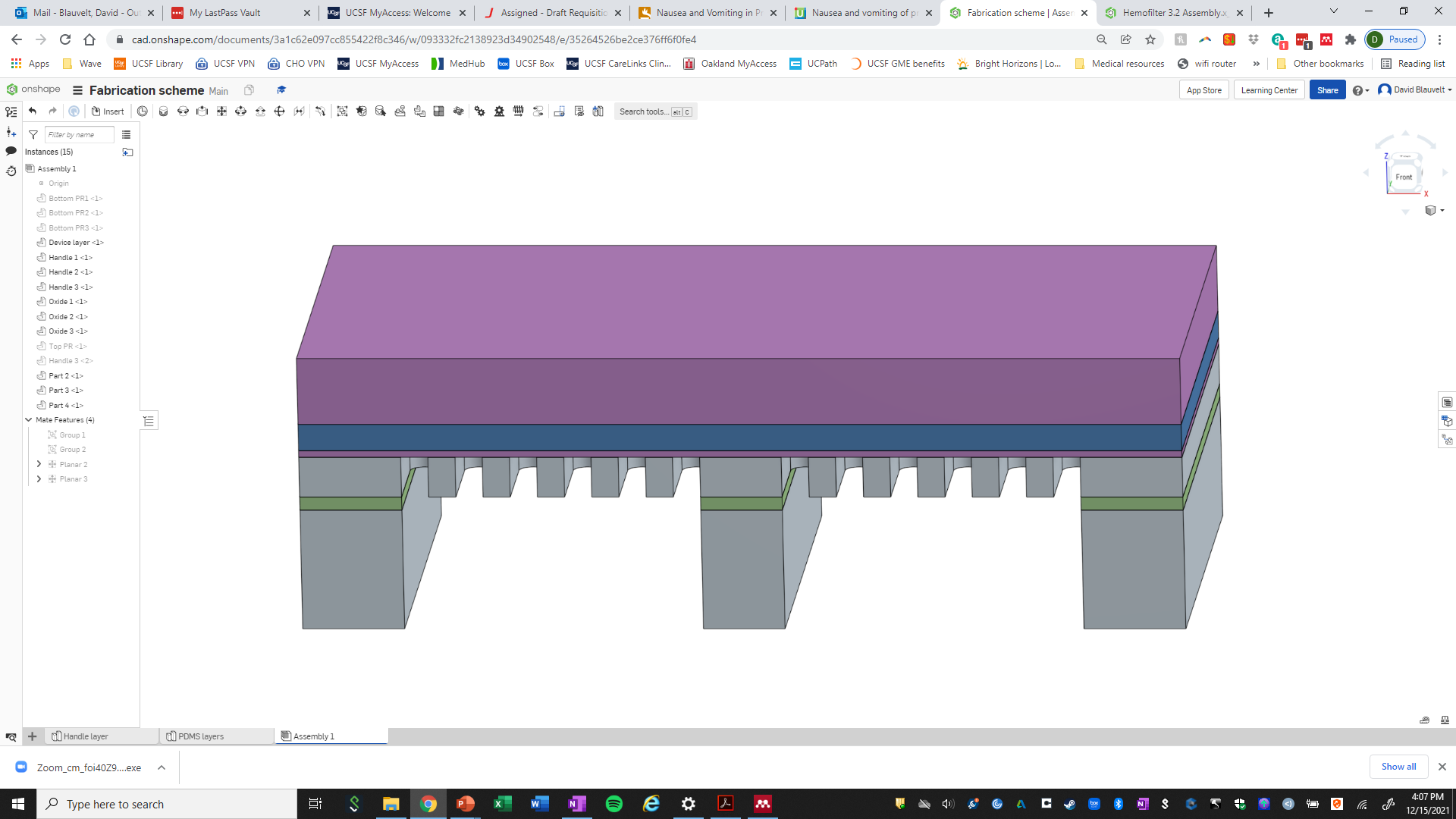

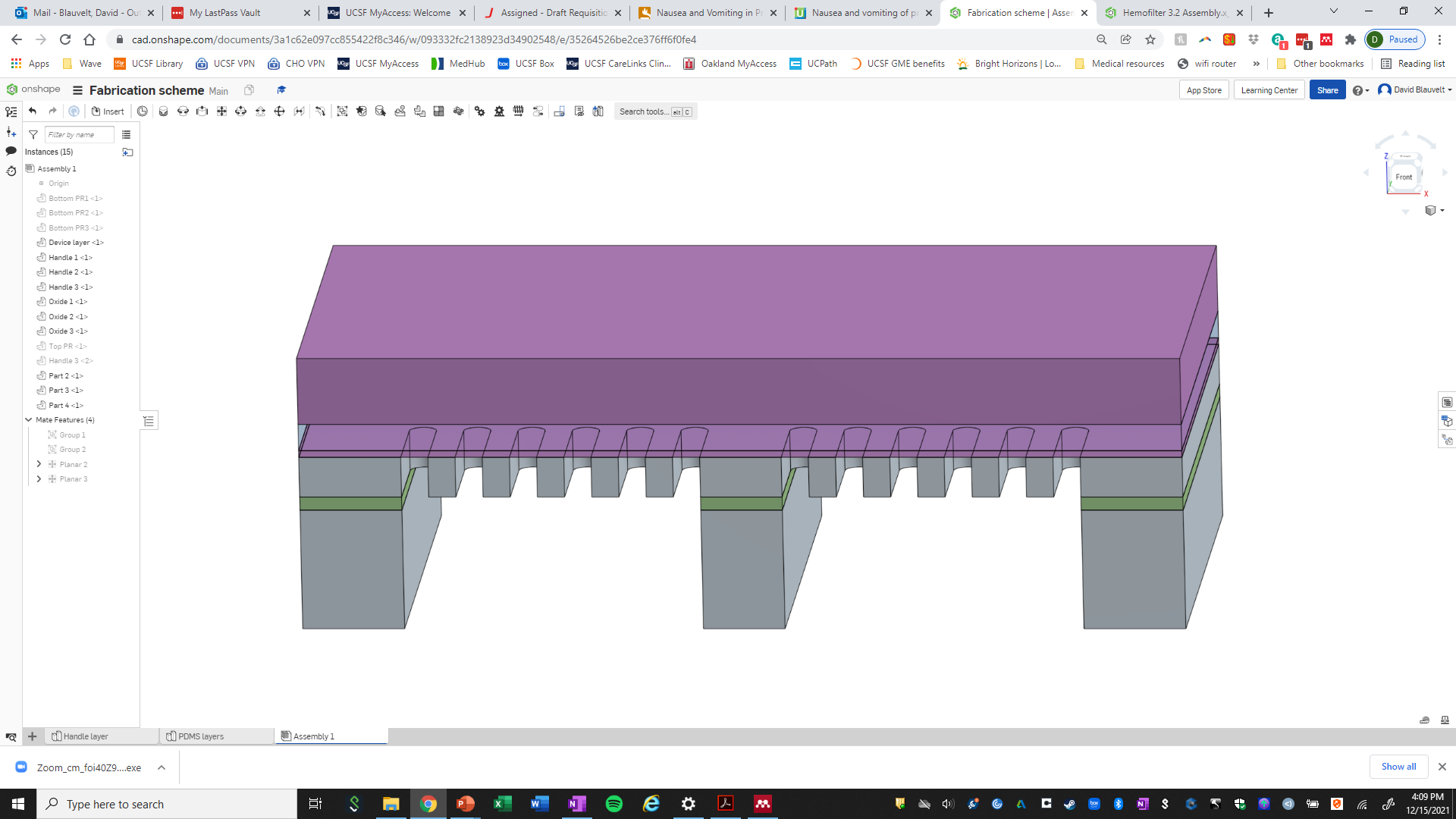

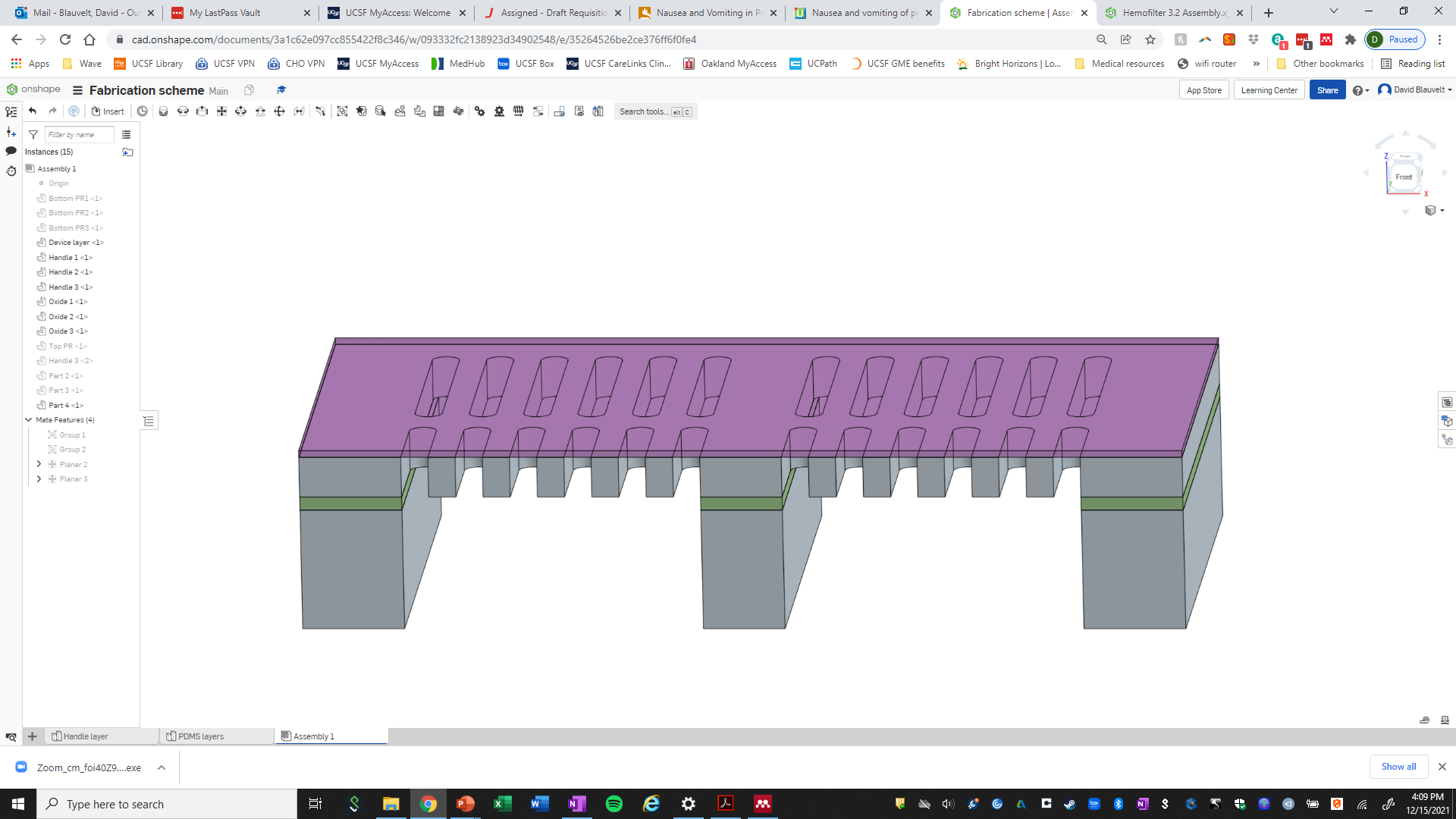


**Figure S1:** Composite silicone membrane fabrication. a) Silicon on insulator (SOI) is etched using a photoresist pattern and deep reactive ion etching (DRIE). Microscopic pores are etched on the top (“pore”) side and large windows are etched on the bottom (“window”) side. The buried oxide insulator layer is dissolved using hydrofluoric acid, connecting the pores to the large windows. b) The thin (5 μm) polydimethylsiloxane (PDMS) polymer layer is spin coated on top of a sacrificial PDMS handle layer, separated by another thin layer of polyvinyl alcohol (PVA). c) The final composite membrane is produced by oxygen plasma bonding the thin PDMS layer to the pore side of the etched SOI component. The sacrificial layers are then released by dissolving the PVA layer in water.

**Table S2:** Device design parameters

|  | Generation 1 Device  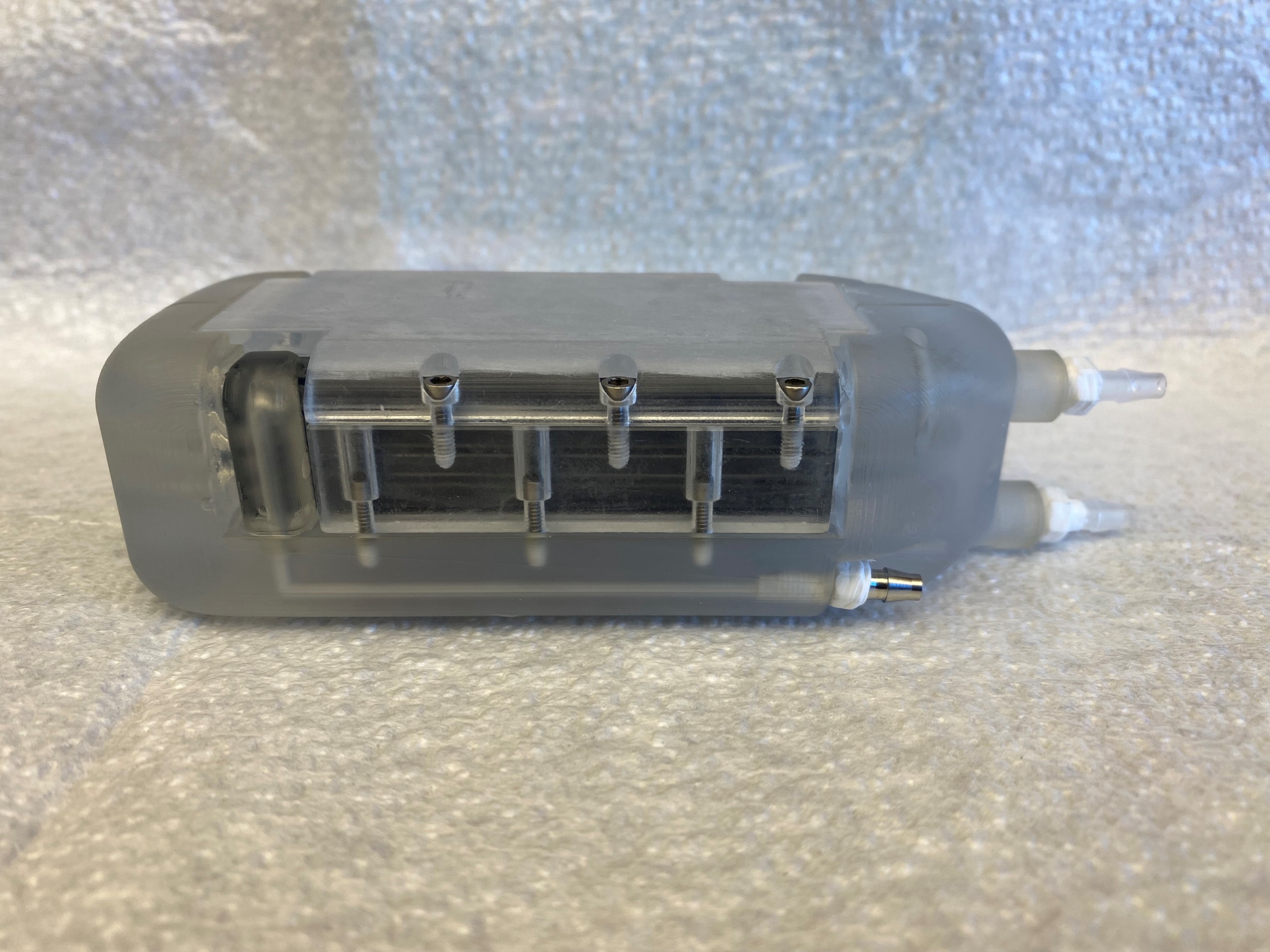 | Generation 2 Device *  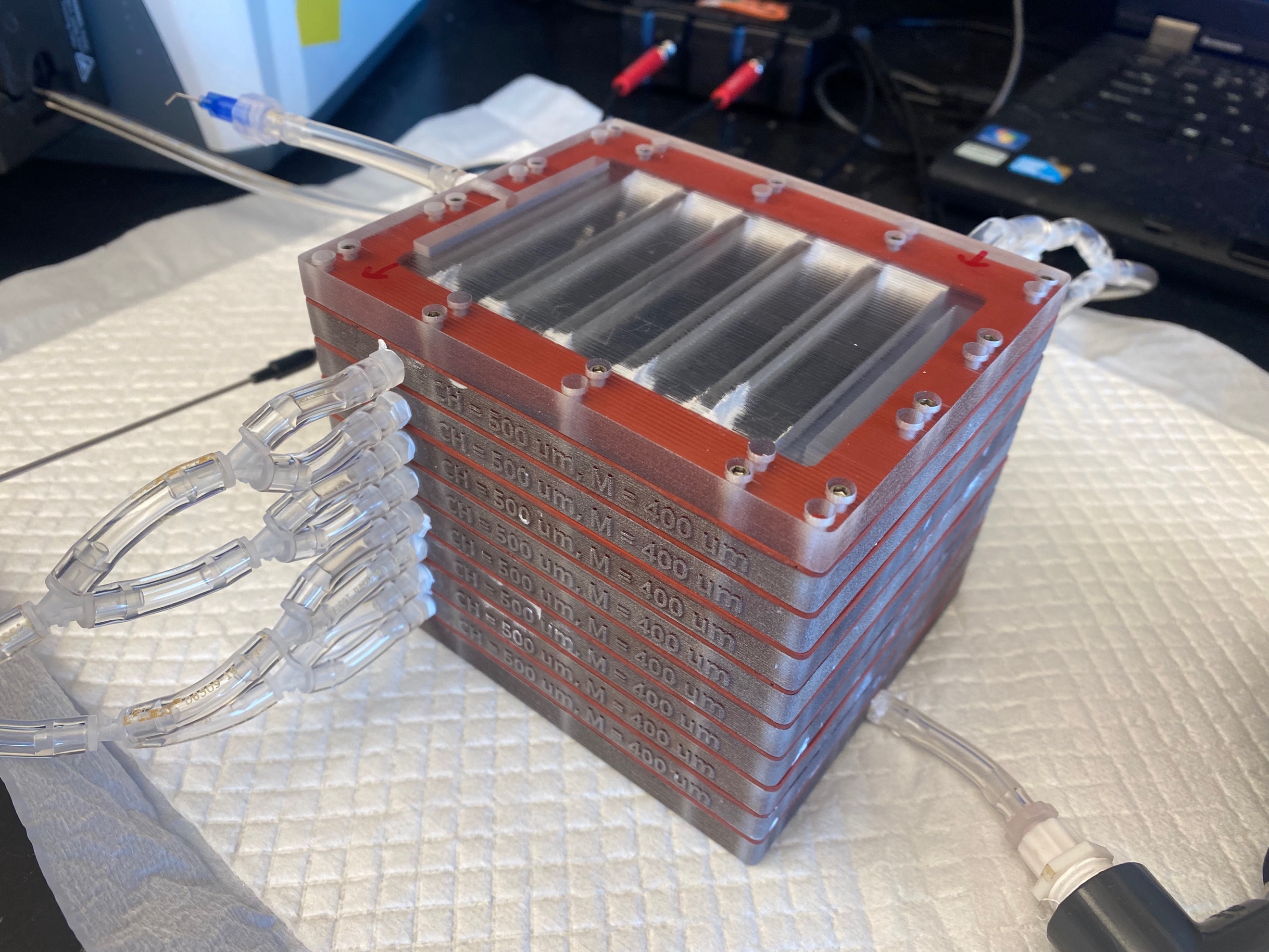 |
| --- | --- | --- |
| Channel Dimensions  Width  Length  Height | 26 mm  65 mm  2 mm | 6 mm  65 mm  0.5 mm |
| Priming Volume | 26 mL | 12 mL (without manifold)  15 mL (with manifold) |
| Membrane surface area | 58 cm^2^ | 192 cm^2^ |
| Blood contacting surface area | 285 cm^2^ | 473 cm^2^ |

* Parameters listed for largest 8-layer device

|  | **10 mL/min** | **20 mL/min** |
| --- | --- | --- |
| **Inlet sO2 (%)** | 82.3 ± 0.8 | 79.3 ± 0.8 |
| **Inlet pO2 (mmHg)** | 46.5 ± 1.1 | 42.4 ± 0.8 |
| **Outlet sO2 (%)** | 87.1 ± 0.7 | 83.3 ± 0.7 |
| **Outlet pO2 (mmHg)** | 53.0 ± 1.7 | 47.0 ± 1.3 |

**Table S3:** Generation 1 Device data

Sweep gas: Unpressurized 100% oxygen.

Values reported as mean ± SEM

**Figure S2:** Scatter plot of MATLAB mass transfer model prediction of the oxygen partial pressure at the outlet versus the measured oxygen partial pressure. Each marker represents a separate experiment with varied conditions, including variable flow, channel dimensions, sweep gas pressure, and inlet partial pressure of oxygen. Dashed line represents perfect alignment between the model predicted and measured partial pressure. Linear regression (solid line) of the data shows excellent agreement between predicted and measured data (slope = 0.97, S_y.x_ = 5.2, r^2^ = 0.99).

**Table S4:** Generation 2 Device data

|  | **10 mL/min** | **60 mL/min** | **70 mL/min** | **80 mL/min** |
| --- | --- | --- | --- | --- |
| **Inlet sO2 (%)** | 71.8 ± 1.6 | 72.7 ± 0.2 | 81.7 ± 0.3 | 83.3 ± 1.1 |
| **Inlet pO2 (mmHg)** | 37.8 ± 1.1 | 38.7 ± 0.2 | 45.7 ± 0.3 | 45.3 ± 0.6 |
| **Outlet sO2 (%)** | 78.0 ± 2.9 | 75.2 ± 0.3 | 84.7 ± 0.4 | 87.5 ± 1.1 |
| **Outlet pO2 (mmHg)** | 43.8 ± 3.3 | 40.2 ± 0.3 | 49.5 ± 0.6 | 49.8 ± 0.8 |

Sweep gas: 100% oxygen at 2 PSI.

Values reported as mean ± SEM.


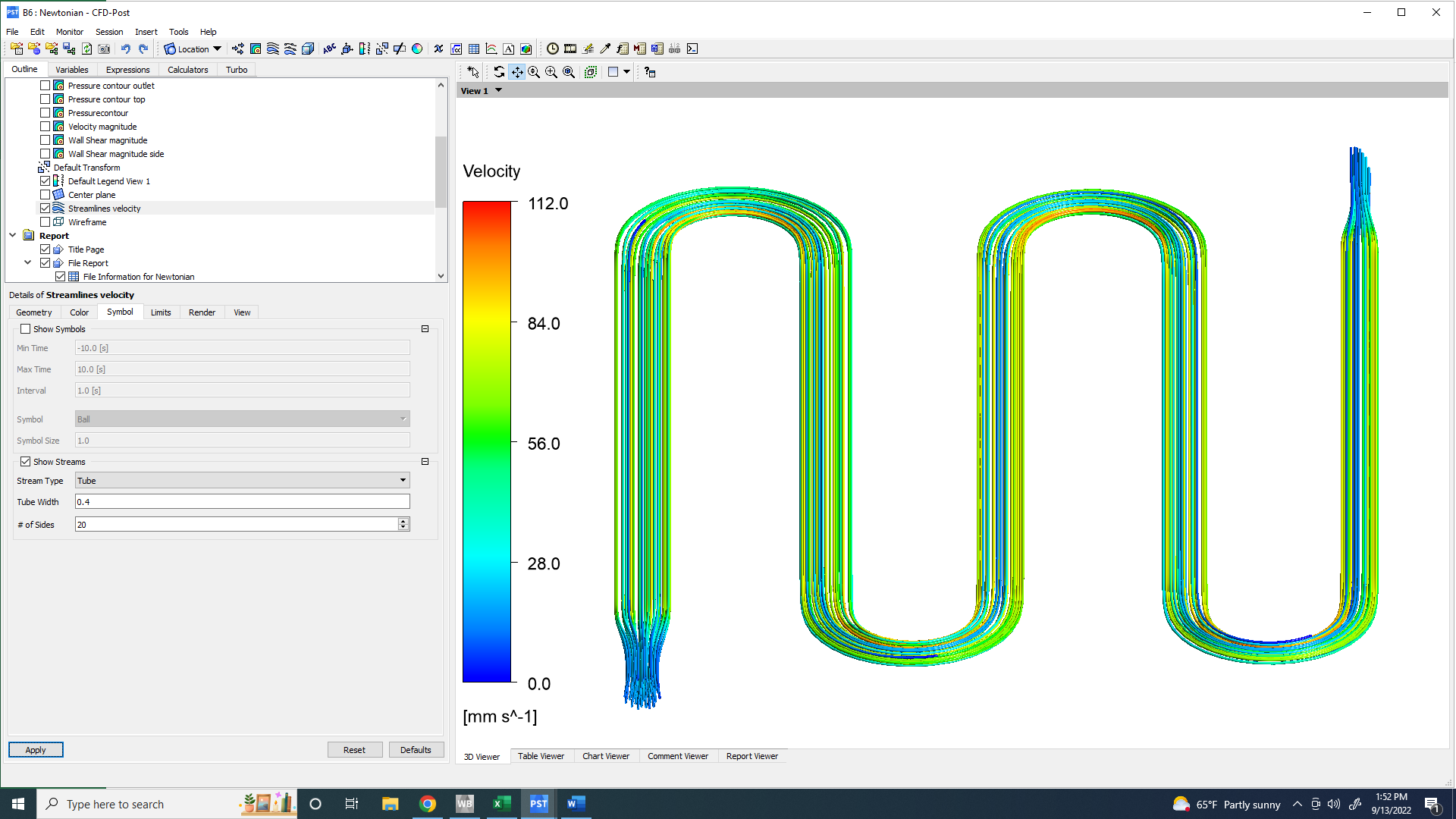


0 28 56 84 112

Velocity (mm/s)


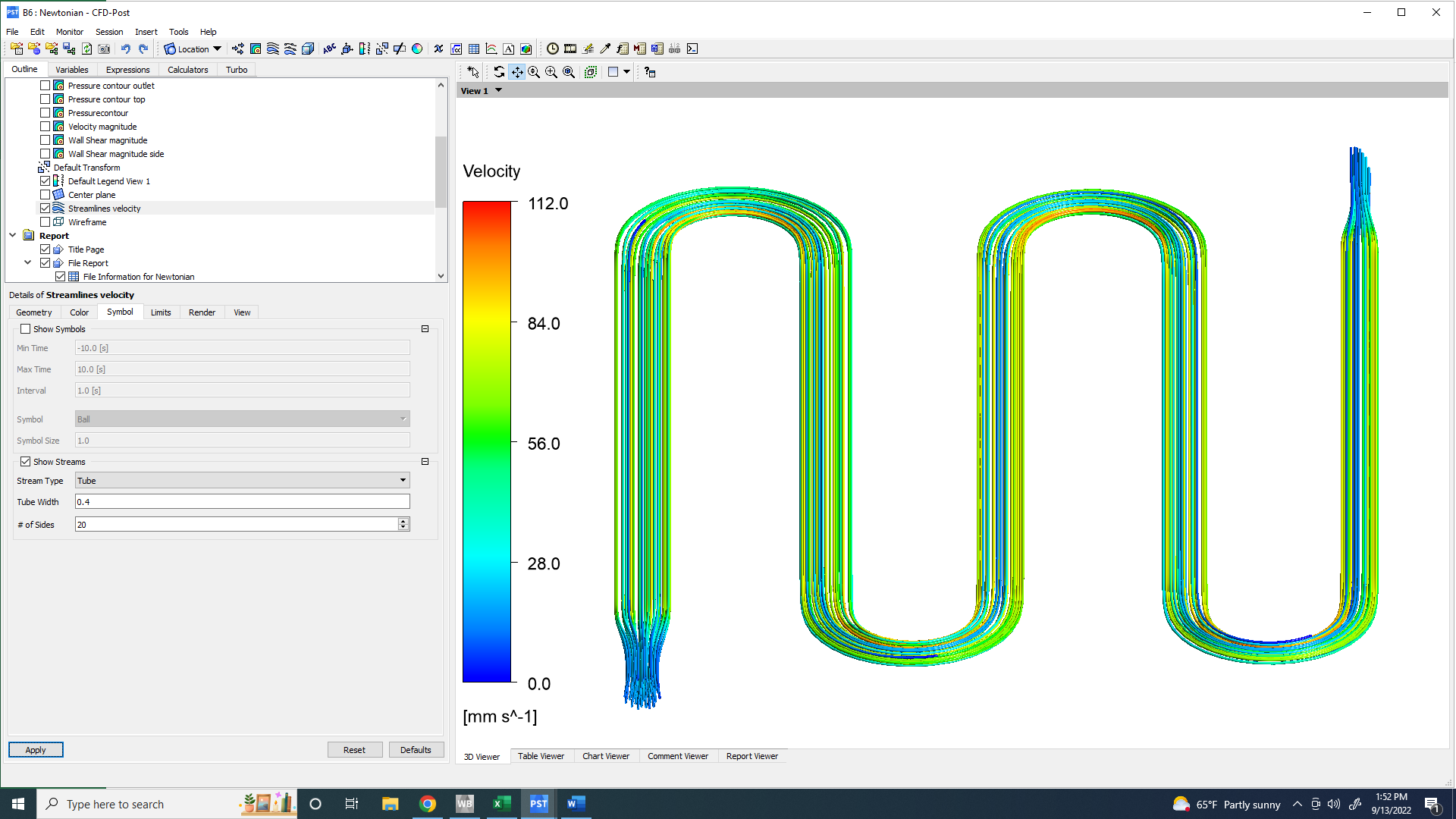


**Table S5:** Computational Fluid Dynamics and Mass Transfer Modeling Constants

**Figure S3:** Computational fluid dynamics (CFD) modeling – streamline analysis. A total of 100 streamlines were generated from equally spaced seeds at the inlet. Streamlines were followed through the flow path to ensure no areas of recirculation, stasis, or turbulence.

| Variable | Value | Units |
| --- | --- | --- |
| **Blood (Fetal Hemoglobin)**  Dynamic Viscosity  Density  Oxygen Diffusivity  Hemoglobin Concentration  P50  Cooperative Binding Factor | 3.5 × 10^-3^  1060  1.97 × 10^-5^  15  18.4  2.8 | Pa · s  kg/m^3^  cm^2^/s  g/dL  mmHg |
| **PDMS**  Density  Oxygen Diffusivity | 997  309.5 | kg/m^3^  Barrer |
| **Testing Conditions**  Temperature  Inlet Oxygen Partial Pressure  Inlet Pressure (Umbilical Artery Pressure)  Sweep Gas Pressure  Blood Flow Rate | 37  18  20  5  200 | Celsius  mmHg  mmHg  PSI  mL/min |
